# Simulated manned Mars exploration: effects of dietary and diurnal cycle variations on the gut microbiome of crew members in a controlled ecological life support system

**DOI:** 10.7717/peerj.7762

**Published:** 2019-09-26

**Authors:** Hai-Sheng Dong, Pu Chen, Yan-Bo Yu, Peng Zang, Zhao Wei

**Affiliations:** 1 Department of Chemistry, Beijing Key Laboratory of Microanalytical Methods and Instrumentation, MOE Laboratory of Bioorganic Phosphorus Chemistry & Chemical Biology, Tsinghua University, Beijing, China; 2 State Key Lab of Space Medicine Fundamentals and Application, Key Laboratory of Space Nutrition and Food Engineering, China Astronaut Research and Training Center, Beijing, China; 3 SPACEnter Space Science and Technology Institute, Shenzhen, China

**Keywords:** Manned Mars exploration, 16S gene sequencing, Controlled Ecological Life Support System Environment, Alpha diversity, Core microbiome, Longitudinal variation, Biomarker screening, Crewmembers, 25-hydroxyvitamin D

## Abstract

**Background:**

Changes in gut microbiome are closely related to dietary and environment variations, and diurnal circle interventions impact on human metabolism and the microbiome. Changes in human gut microbiome and serum biochemical parameters during long-term isolation in a controlled ecological life support system (CELSS) are of great significance for maintaining the health of crewmembers. The Green Star 180 project performed an integrated study involving a four-person, 180-day duration assessment in a CELSS, during which variations in gut microbiome and the concentration of serum 25-hydroxyvitamin D, α-tocopherol, retinol and folic acid from the crewmembers were determined.

**Results:**

Energy intake and body mass index decreased during the experiment. A trade-off between Firmicutes and Bacteroidetes during the study period was observed. Dynamic variations in the two dominant genus Bacteroides and Prevotella indicated a variation of enterotypes. Both the evenness and richness of the fecal microbiome decreased during the isolation in the CELSS. Transition of diurnal circle from Earth to Mars increased the abundance of Fusobacteria phylum and decreased alpha diversity of the fecal microbiome. The levels of serum 25-hydroxyvitamin D in the CELSS were significantly lower than those outside the CELSS.

**Conclusions:**

The unique isolation process in the CELSS led to a loss of alpha diversity and a transition of enterotypes between Bacteroides and Prevotella. Attention should therefore be paid to the transition of the diurnal circle and its effects on the gut microbiome during manned Mars explorations. In particular, serum 25-hydroxyvitamin D levels require monitoring under artificial light environments and during long-term space flight. Large-scale studies are required to further consolidate our findings.

## Introduction

Long-term manned spaceflight and the development of extraterrestrial planet settlement measures are of great interest in aerospace technology. An effective approach to realize this goal is the establishment of a controlled ecological life support system (CELSS) that provides all the essential living requirements, such as food, oxygen, and water through continuous regeneration (Nelson, Dempster & Allen, 2008). The CELSS is also termed as a bio-regenerative life support system, biological life system, or third-generation life support system, and is a closed micro-ecological circulation environment that is artificially constructed based on the characteristics of the space environment (Guo et al., 2008). In this system, plants provide food and oxygen for heterotrophic organisms (humans and animals) through photosynthesis, and convert carbon dioxide and other waste from heterotrophic organisms into useful products, constituting the carbon and oxygen cycles of the system (MacElroy & Bredt, 1984). Plants participate in the system’s water purification cycle through root absorption and leaf transpiration. Microbes degrade, mineralize, and regenerate inedible parts of plants, occupant excrement, and domestic wastewater; providing water and nutrients for plants and food for the inhabitants. This completes the food recycling process and creates a closed-circuit ecosystem that is continually renewed by plants, animals, microorganisms, humans, and the necessary organic and inorganic environment (Degermendzhi & Tikhomirov, 2014). In current short or medium-term manned space activities, aerospace foods are periodically transported from the ground to space stations through a cargo spaceship, which is technically and economically feasible. However, for the future establishment of long-term manned space missions, including Lunar or Mars bases, the existing supply mode of food systems will shift from on-earth supplies to biologically regenerated food systems in situ (Meinen et al., 2018). CELSS is an important environmental model for long-term manned space station missions and human activities at extra-territorial bases. Studies have shown that the frequency of health problems increase in a closed environment, including physical and psychological issues (Stein, 2001). For manned space flight studies, the major drawbacks include limited spacecraft space, which makes large scale studies on numerous individual subjects unfeasible (Lang et al., 2017). Repeated measurements of individuals during longitude research, particularly for twin samples, can serve as an efficient study method (Garrett-Bakelman et al., 2019). Studies investigating the temporal dynamics of the gut microbiome in six individuals sharing a confined environment during a 520-day ground-based space simulation indicated the importance of maintaining a health-promoting, mutualistic microbiome environment (Mardanov et al., 2013; Turroni et al., 2017).

Gut microorganisms are the largest micro-ecological systems in the human body encoding ≥100 times the coding genes of humans (Eckburg et al., 2005). There are at least 160 dominant microorganisms in each individual, more than 90% of which belong to *Bacteroidetes* and *Firmicutes* (Shafquat et al., 2014). In recent years, the continuous development and application of new technologies such as high-throughput sequencing and metagenomics have revealed new perspectives for understanding human life activities (Yibin, 2014; Yu et al., 2017). Gut microorganisms profoundly affect human health by participating in nutrient metabolism and immune system development, and have emerged as new targets for precision medical interventions (Botero et al., 2016; Saraf et al., 2017). Changes in the internal and external environment of the human body can affect the species, structure, and function of the gut microbiome (He et al., 2015; Lin et al., 2015). Through constant adaptation and regulation, the flora, host, and environment together maintain physiological balance and health (Botero et al., 2016; He et al., 2015).

The lower gastrointestinal tract of humans hosts trillions of diverse microbial communities, each with unique and extensive metabolic capacities, producing a variety of nutrients including B vitamins, and vitamin K (LeBlanc et al., 2013). The gut microbiome also contribute to human metabolism and digestion which have profound effects on the pathogenesis of metabolic syndrome (Ramakrishna, 2013). The low levels of natural light in a manned spacecraft has negative effects on serum vitamin D levels, whilst changes in the intestinal microbiome influence vitamin D metabolism (Bora et al., 2018). Furthermore, evidence suggests that consistent long-term physical and psychological stress damage in an isolated weightlessness environment leads to decreased serum vitamin E and vitamin A levels, and a loss of antioxidants (Smith et al., 2005). Meanwhile, variations in normal human daily behaviors (wakefulness) and rest (sleep) rhythmicity or diurnal rhythmicity have been reported to impact metabolism, the microbiome and circadian clocks (Nobs, Tuganbaev & Elinav, 2019; Reinke & Asher, 2019). Since the dynamic changes of the microbiome influences serum biochemistry for crewmembers living in CELSS, studies have focused on their responses during simulated Mars exploration missions for a 180-day period. In this study, we hypothesized that during the 180 days in the CELSS, dietary changes, the isolated environment and scheduled diurnal cycles may lead to fluctuations in the gut microbiome and specific serum vitamins. We performed 16S gene sequencing technology to analyze longitudinal changes in the human gut microbiome during 180-day long-term residence in CELSS. Blood samples were collected for the analysis of serum 25-hydroxyvitamin D, α-tocopherol, retinol and folate. The experiments were designed to simulate changes in the intestinal microbiome structure during the long-term residency of astronauts in the controlled environment of extraterrestrial planets. Our findings provide a basis for promoting the intestinal and nutritional health of astronauts during long-term space flight missions.

## Materials and Methods

### Green Star 180 project

**Figure 1:**
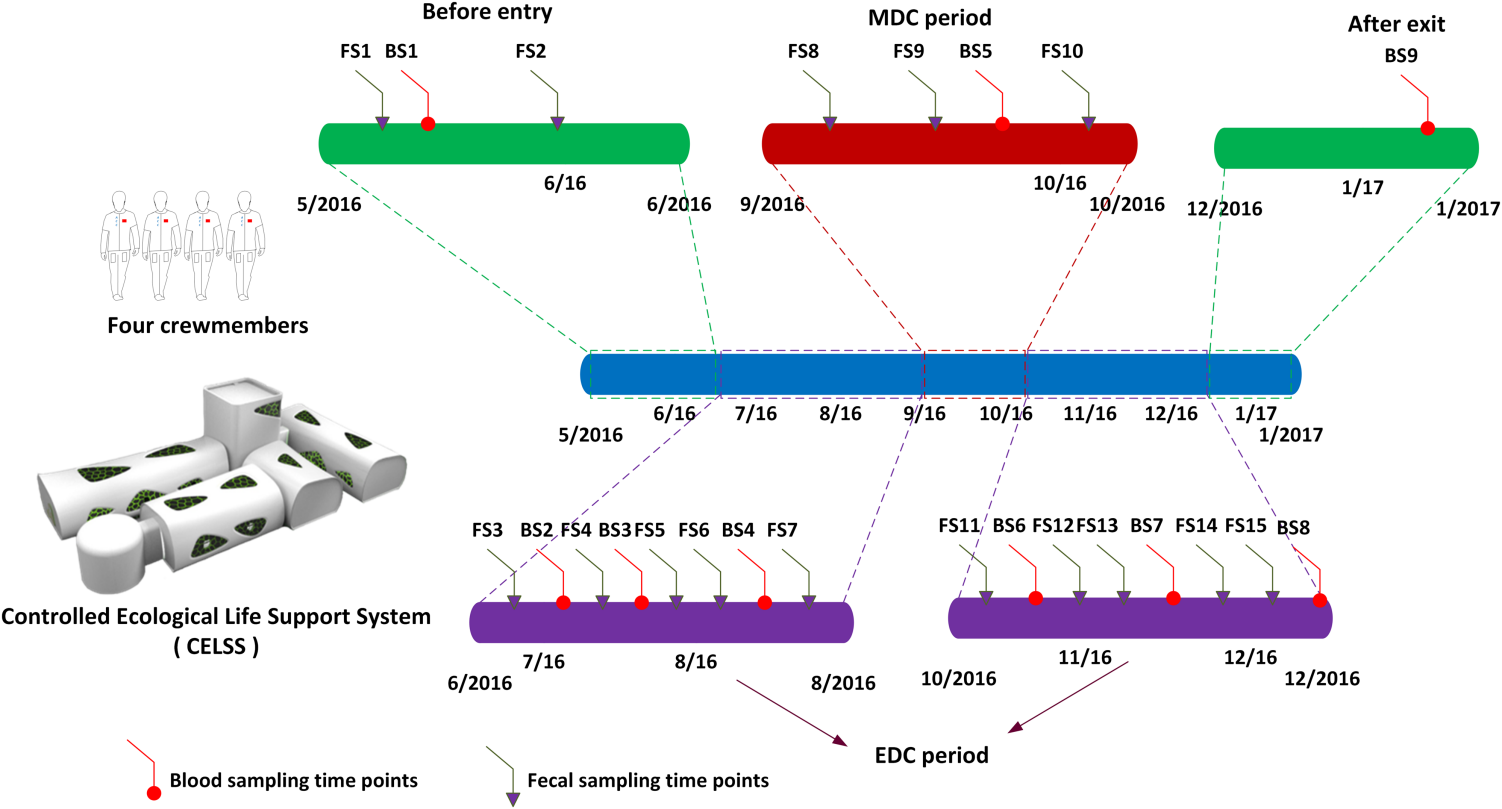
Scheduled fecal and blood sampling time points. MDC, Mars diurnal circle; EDC, Earth diurnal circle. FS1–FS15 refers to fecal sampling time points. BS1–BS9 refers to blood sampling time points.

Four volunteers (three males and one female, aged 26–36 years) that passed rigorous health examinations and had comparable body mass indexes similar to those of astronauts (BMI = 18.6–24.6 kg/m^2^) were screened as crewmembers for the Green Star 180 project (GS-180). The Ethics Committee of the Astronaut Research and Training Center of China approved the project (ethics approval number: Szsisc EA201601). Crewmembers were healthy with no digestive system disease, alcohol abuse, diabetes or other problems affecting the gut microbiome, and were compatible in terms of dietary habits. No antibiotics or probiotics was used within 3 months of the trial. Various activities were scheduled for the crewmembers such as system monitoring and recording, biochemistry experimental projects, crop cultivation and harvesting, solid waste incineration, equipment operating and maintenance and physical training. Crewmembers were familiar with the trial process and mastered each professional technique. The CELSS consisting of eight modules: crew module I, crew module II, the CELSS module, the resource module, plant module I, plant module II, plant module III, and plant module IV, in which specific crops were planted are shown in Table S1. The living environment in the CELSS was suitable for human physiological requirements with a temperature 23–25 °C, humidity 46–77% R.H., an atmospheric pressure of 81.3–104.3 kPa, O_2_ 18–26% and CO_2_ ≤ 0.8%. The foodstuffs provided were served as prepackaged food outside the CELSS and freshly prepared foods supplied by the self-producing plants (including five crops, 16 vegetable plants, and two fruit plants) grown in the CELSS. Recipes for the four crewmembers during their 180-day residence in the CELSS are shown in Table S2. The shelf life of the space foods, ingredients and seasoning provided covered the task duration. The dietary design for the crewmembers was in accordance with the customs of the Chinese population including three meals a day, namely breakfast, lunch and supper. Complementary foods were provided as snacks or refreshments. The main compositions of the diet included carbohydrates, protein, fat, and dietary fiber which were measured through the calculation of nutrient composition in the edible portion of specific food items through reference to the authoritative handbook: China Food Composition recommended by the Chinese Nutrition Society (Yang & Pan, 2009). The specific calculation strategy was in accordance with recently published references (Hao et al., 2018; Kirchhoff, 2002). To simulate the manned Mars exploration mission, both Earth (24 h) and Martian diurnal cycles (MDCs) (24 h 40 min) were employed. The Earth diurnal cycle (EDC) was adopted after entering the CELSS from day 1 to day 72 and day 110 to day 180, whilst days 73 to 109 used the MDC through time information control, scheduled light exposure (150 Lx white light LED) and the adjustment of waking and rest rhythmicity. Fecal samples collected in sterile sample collection tubes are shown in Fig. 1. The prescribed time points were as follows: 30 days before entering the CELSS, 15 days before entering the CELSS, on days 2d, 15d, 30d, 45d, 60d, 75d, 90d, 105d, 120d, 135d, 150d, 165d and 175d after entering the CELSS. All the fecal samples were collected in the morning into clean stool collection tubes at scheduled time points, sealed and transferred to a −80 °C freezers prior to analysis.

### Microbial DNA extraction, Illumina MiSeq sequencing and data analysis

After thawing at 4 °C, DNA was extracted from the fecal samples using the cetyl trimethylammonium bromide method (Hamilton et al., 2013; Wu et al., 2016) to remove impurities including proteins and polysaccharides. Total DNA was extracted from 150 mg of fecal samples using commercially available DNA stool MiniKits (Qiagen, Hilden, Germany) and stored at −80 °C until analysis. Forward PCR primers: 338F: 5′ACTCCTACGGGAGGCAGCAG3′; and reverse primers were 806R: 5′GGACTACHVGGGTWTCTAAT3′ from the V3–V4 conserved region of 16S rDNA. PCR reactions to the target regions were performed using the 96 well AB9902 PCR system in 50 μL reactions: 25 μL KOD FX Neo Buf, one μL KOD FX Neo, 1.5 μL of forward and reverse primers (10 μM), 10 μL dNTP (two mM), five nL (40–60 ng) template DNA, up to 50 μL in ddH_2_O. Reaction sequence conditions were as follows: initial denaturation at 95 °C for 5 min, 15 cycles of denaturation at 95 °C for 1 min, annealing at 50 °C for 1 min, and elongation at 72 °C for 1 min. Final elongation at 72 °C for 7 min. PCR products were sequenced using paired-end sequencing platforms on Illumina Hiseq 2500. Raw sequence data obtained were stored in the NCBI online public database with accession number PRJNA557020.

Raw tag acquisition: sequence reads were merged using FLASH v1.2 software with a minimum overlap length of 10 bp and a maximum mismatch ratio of 0.2 to obtain Raw Tags. Clean tag acquisition: clean tags were obtained by filtering merged raw tags with Trimmomatic v0.33 software (threshold: 50 bp). Effective tag acquisition: chimera sequences were removed using UCHIME v2.4 software. Operational taxonomy units (OTU) analysis: OTU refer to clusters of (uncultivated or unknown) microbial organisms at different taxonomic levels grouped by sequence similarities in specific marker genes. OTU categorization was performed at thresholds ≥97% using USEARCH software version 10.0. Taxonomic annotation of the OTUs was accomplished by matching all OTU tags to the SILVA (128) database (http://www.arb-silva.de).

### Determination of serum 25-hydroxyvitamin D, α-tocopherol, retinol and folate

All crewmembers were asked to collect blood samples according to prescribed time points: 30 days before entry, on days 2d, 30d, 60d, 90d, 120d, 150d, and 180d, and 30 days after exiting the CELSS. All blood samples were collected in the morning using vacuum blood collection tubes, and centrifuged at 4,000×*g* for 15 min (4 °C). Supernatants were transferred to a −80 °C freezer for further analysis. The determination of serum 25-hydroxyvitamin D, α-tocopherol and retinol was performed using conventional High Performance Liquid Chromatography (HPLC) quantitative analysis methods (Rock et al., 1999). Serum folate determinations were performed through competitive chemiluminescent enzyme immunoassay methods (Cumurcu, Sahin & Aydin, 2006; Kullak-Ublick et al., 2016).

### Statistical analysis

Data analysis and visual explorations were performed using the online statistical analysis platform Calypso Version 8.84 (Zakrzewski et al., 2016) and GraphPad Prism 5.0. Alpha diversity was performed by straight observational diversity metrics as evenness and richness. Multivariate data visualization and repeated measurement ANOVA assessments were used to measure the accumulation factor of the environment on crewmembers with *p*-value cutoff 0.05.

## Results

### Longitudinal variations of gut microbiome

**Figure 2:**
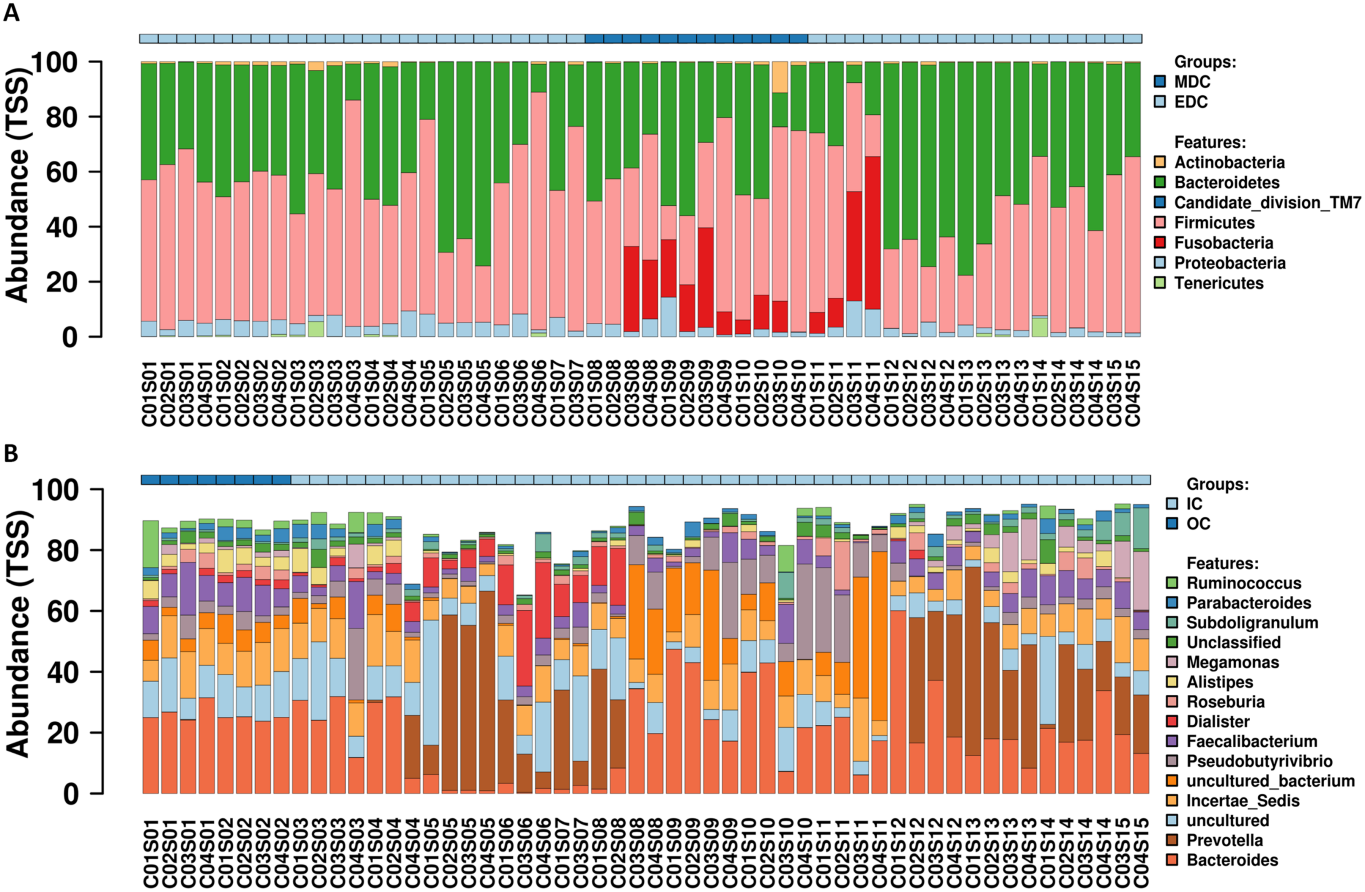
Relative abundance variation profiling of fecal microbiota. (A) At phylum level. (B) At genus level. Sequenced C0#S01 to C0#S15 in Fig. S1 refer to data from fecal samples for crewmember 0# 30 days before entering the CELSS, 15 days before entering the CELSS, and 2d, 15d, 30d, 45d, 60d, 75d, 90d, 105d, 120d, 135d, 150d, 165d and 175d after entering the CELSS. MDC, Mars diurnal cycle; EDC, Earth diurnal cycle; OC, period when crewmembers were out of the CELSS (before entry); IC, period when the crewmembers were in the CELSS.

A total of 54 fecal samples passed the quality control and were identified by next-generation sequencing of the V3–V4 hypervariable region of the 16S rRNA gene, with a total of 3,345,159 high-quality sequence reads produced (mean per sample, 61,947; range, 57,791–65,818). Data missing at certain sampling time points was attributed to that certain raw data reads obtained from the HiSeq platform did not pass the quality control. Reads were clustered into filtered OTUs at 97% sequence similarity. OTU annotations were performed using SILVA, and 357 OTUs were identified according to previously described normalization strategies (Singh et al., 2012; Wang et al., 2009; Wong, Chaston & Douglas, 2013). Relative abundance profiling at the phylum and genus level for each crewmember was visualized as shown in Fig. 2, and relative abundance profiling at the order, family and species levels are shown in Fig. S1. Core microbiomes for each crewmember at the phylum and genus level (top 10) are shown in Fig. S2. The results showed dominant microbiomes at the phylum level in fecal samples from the four crewmembers were Firmicutes, Bacteroidetes, Proteobacteria, Actinobacteria and Fusobacteria. Dynamic tradeoff of the two most abundant fecal microbiomes Firmicutes and Bacteroidetes were observed during the process (shown in Fig. S3). Dominant fecal microbiomes at the genus level in fecal samples from the four crewmembers were Bacteroides, Prevotella, Incertae_Sedis, Faecalibacterium, Pseudobutyrivibrio, Alistipes, Dialister and Roseburia. We also observed a dynamic tradeoff of Bacteroides and Prevotella during the study period (Fig. S4).

### Diversity analysis

**Figure 3:**
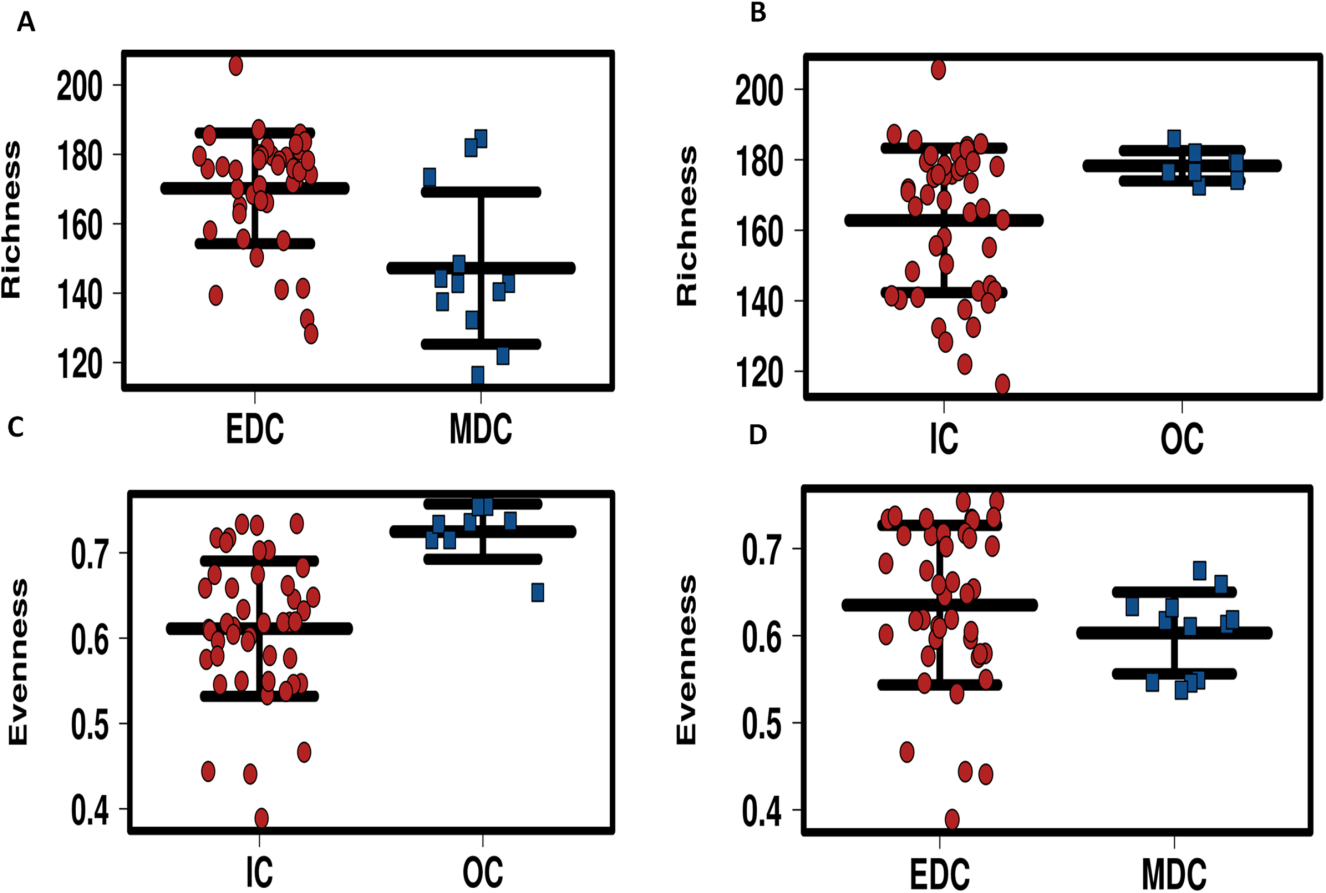
Profiling of alpha diversity for fecal microbiome. (A) Richness comparison between EDC and MDC. (B) Richness comparison between IC and OC. (C) Evenness comparison between IC and OC. (D) Evenness comparison between EDC and MDC. MDC, Mars diurnal cycle; EDC, Earth diurnal cycle; OC, period when crewmembers were out of the CELSS (before entry); IC, period when the crewmembers were in the CELSS.

Alpha diversity of microbiomes reflects the general richness and evenness of the species communities in the habitats. As shown in Fig. 3 and Fig. S5, compared to the fecal microbiome outside the system, both evenness and richness showed significant decreases after CELSS admission (*p* < 0.01). Meanwhile, the richness of the fecal microbiome decreased when the crewmembers performed MDC schedules, which recovered to their original status when the EDC schedule was resumed.

### Biomarker screening

Repeated ANOVA analysis was used to explore fecal microbiome at the phylum and genus level (*p* < 0.05). Results are shown in Table 1. The level of Fusobacteria, Paraprevotella, Phascolarctobacterium, Pseudobutyrivibrio, Bacteroides and Collinsella when the EDC schedule was performed were significantly lower than those of the MDC schedule. The levels of Bacteroidetes, Firmicutes, Prevotella, Alistipes, Megamonas and Sutterella when the EDC schedule was performed were significantly higher than those of when the MDC schedule was performed.

**Table 1:** Fecal microbiota at phylum and genus level with significant differences by repeated measurement ANOVA analysis.

Taxa	*p*-value	Comparison between treatments
	Phylum level	IC vs OC
Firmicutes	0.0082	<
Fusobacteria	0.0099	>
Bacteroidetes	0.0170	>
	Genus level	IC vs OC
Faecalibacterium	0.0001	>
Alistipes	0.0001	<
Lachnospira	0.0004	<
Prevotella	0.0010	>
Ruminococcus	0.0017	<
Incertae_Sedis	0.0045	<
Bacteroides	0.0088	<
Pseudobutyrivibrio	0.0300	>
Butyricimonas	0.0310	<
Parabacteroides	0.0380	<
Subdoligranulum	0.0390	>
Dialister	0.0480	<
	Phylum level	EDC vs MDC
Fusobacteria	0.0003	<
Bacteroidetes	0.0084	>
Firmicutes	0.0170	>
	Genus level	EDC vs MDC
Paraprevotella	0.0000	<
Phascolarctobacterium	0.0002	<
Pseudobutyrivibrio	0.0028	<
Prevotella	0.0034	>
Bacteroides	0.0061	<
Alistipes	0.0081	>
Megamonas	0.0120	>
Collinsella	0.0190	<
Sutterella	0.0340	>
	Phylum level	Crewmembers
Fusobacteria	0.0011	C03 > C02 > C04 >> C03
Bacteroidetes	0.0001	C02 > C01 > C03 > C04
Firmicutes	0.0003	C04 > C03 > C01 > C02
Proteobacteria	0.0360	C01 > C03 > C04 > C02
	Genus level	Crewmembers
Bacteroides	0.0005	C02 > C01 > C03 > C04
Prevotella	0.0006	C03 > C04 > C01 > C02
Pseudobutyrivibrio	0.0035	C03 > C01 > C04 > C02
Megamonas	0.0070	C04 > C03 > C02 > C01
Incertae_Sedis	0.0086	C04 > C03 > C01 > C02
Faecalibacterium	0.0180	C04 > C03 > C01 > C02
Dialister	0.0180	C01 > C02 > C04 > C03
Alistipes	0.0210	C02 > C01 > C03 > C04
Subdoligranulum	0.0380	C04 > C03 > C02 > C01
Roseburia	0.0420	C02 > C01 > C04 > C03

**Note:**

MDC, Mars diurnal cycle; EDC, Earth diurnal cycle; OC, period when crewmembers were out of the CELSS (before entry); IC, period when the crewmembers were in the CELSS. C01, C02, C03 and C04 refers to crewmember 01, 02, 03 and 04.

### Dietary variations

**Figure 4:**
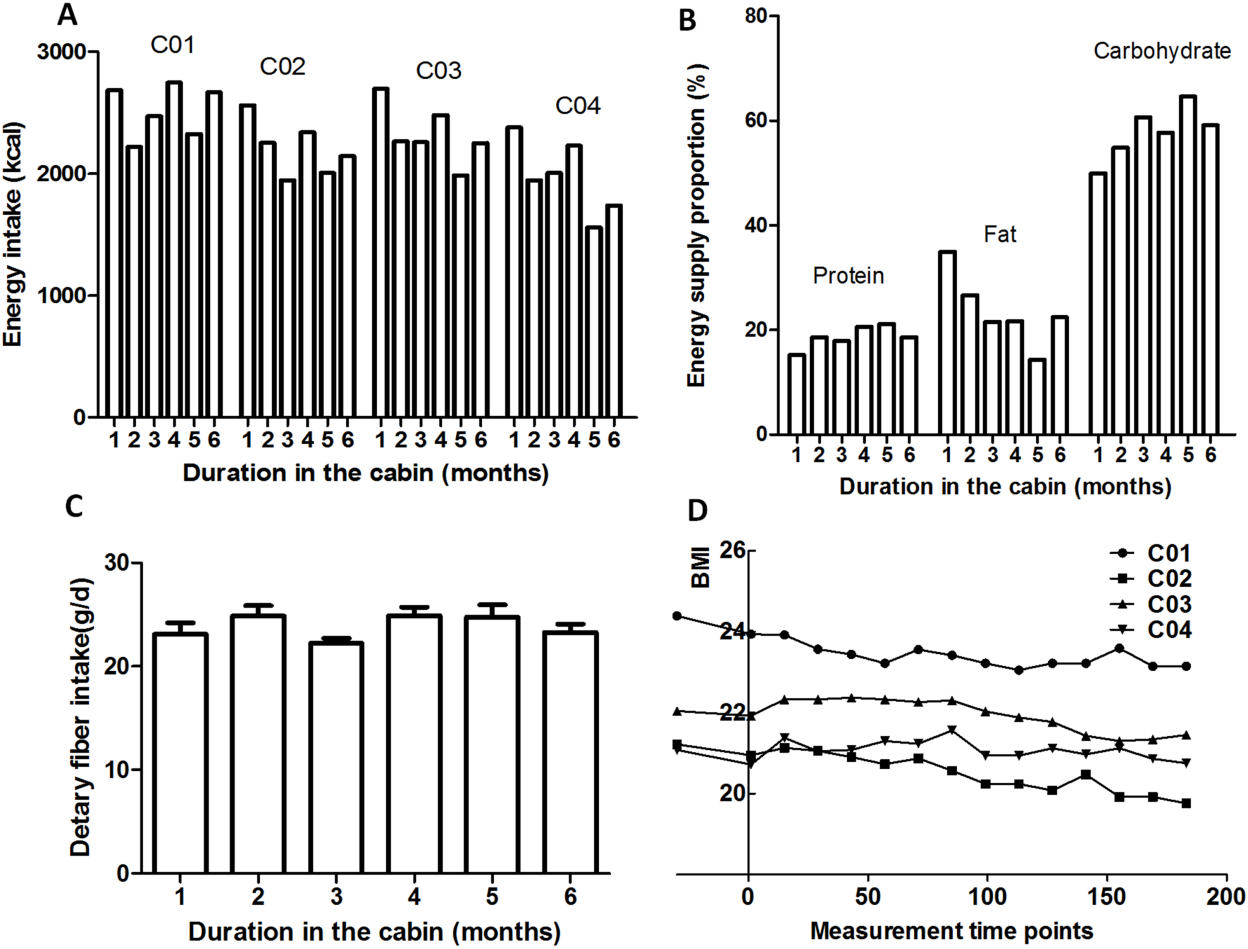
Dietary and BMI variations. (A) Energy intake. (B) Energy supply proportion. (C) Average dietary fiber intake. (D) BMI. C01, C02, C03 and C04 refers to crewmember 01, 02, 03 and 04.

As shown in Fig. 4, decrease of the BMI values of the four crewmembers was accompanied by the reduced energy intake. During the 180-day mission, the controlled eco-surveillance system did not achieve 100% of the required food supply, which was supplemented with pre-packaged food (detailed recipe is shown in a Supplemental File). Due to the higher consumption of prepackaged food compared to fresh food at the beginning of the study, dietary fiber was lower during these stages. The proportions of the three major energy nutrients during dietary intake are shown in Fig. 4. Energy was mainly provided by carbohydrates (48.9–65.5%) which increased over the study period. The energy provided by protein was 14.9–22.4%, which also increased across the duration of the study. The energy provided by fat was 13.6–35.4%, which decreased as the study progressed.

### Variation of the serum 25-hydroxyvitamin D, α-tocopherol, retinol and folate

**Figure 5:**
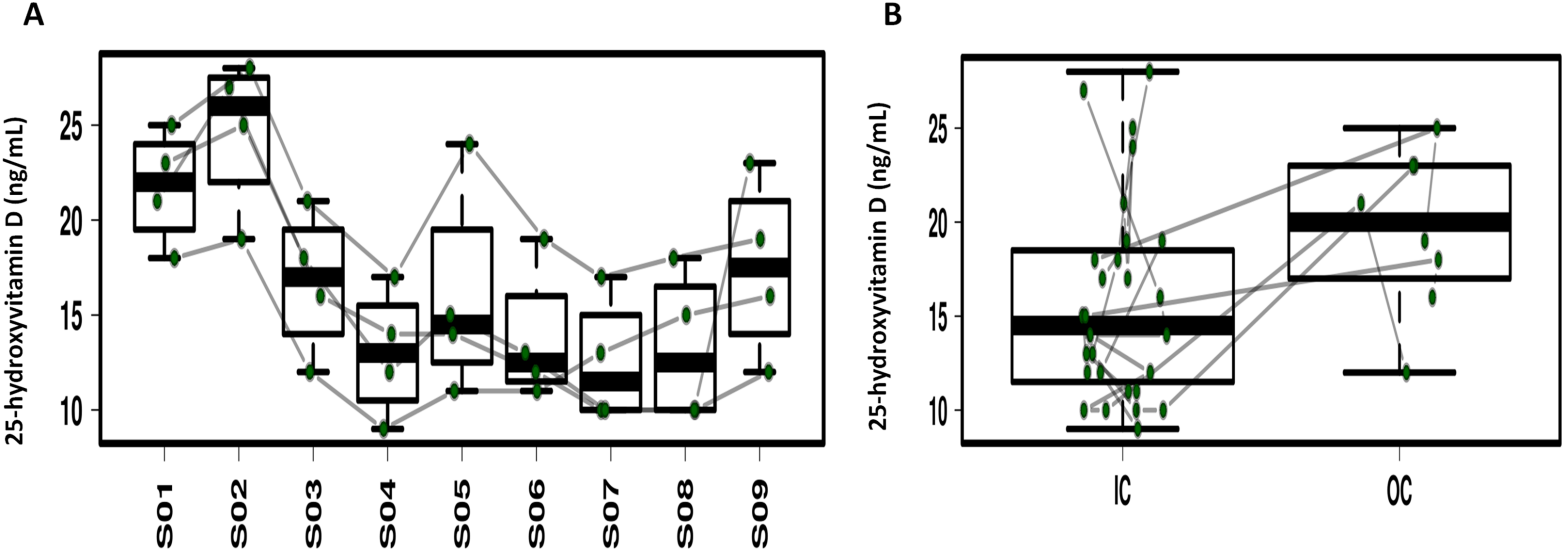
Profiling of the serum 25-hydroxyvitamin D concentrations. (A) Variation during the experiment. (B) Comparison between IC and OC. S01–S09 refers to blood sampling time points: 30 days before entry (S01), on days 2d (S02), 30d (S03), 60d (S04), 90d (S05), 120d (S06), 150d (S07), and 180d (S08), and 30 days after exiting the CELSS (S09).

Repeated ANOVA analysis was used to explore the temporal dynamics of the four serum vitamins (Fig. 5; Fig. S6). Fluctuations were observed for serum α-tocopherol, retinol and folate but the differences were not significant. Serum 25-hydroxyvitamin D in 3/4 of the study participants significantly decreased during CELSS residence, but increased on day 30 after CELSS departure. The levels of serum 25-hydroxyvitamin D in the CELSS were thus lower than those outside the CELSS.

## Discussion

### Enterotypes and diversity variation in the CELSS system

The CELSS included a reliable functional system, including basic environmental regulation, regenerative life support, critical life support measurements and control systems, which could accurately control temperature, humidity, atmospheric pressure and air composition of the enclosed environment. Changes in temperature and atmospheric pressure in the CELSS were minor and identical for all crewmembers (Dai et al., 2017). Studies on host and gut microbiome interactions are complicated by compound environmental factors, such as dietary habits, the host genotype, and occupations, that can lead to drastic variations in gut microbiome. The gut microbiome of the Asian population can be classified into three enterotypes namely: Bacteroides, Prevotella, and Enterobacteriaceae (Arumugam et al., 2011; Liang et al., 2017). In this study, dynamic variations of Bacteroides-Prevotella-Bacteroides-Prevotella were observed (Fig. S4), which may have resulted from a cumulative effect of both the diet and environment (Liang et al., 2017). The variations in diversity showed that changes in the fecal microbiome in the isolated environment were closely related to scheduled diurnal cycles. The host can influence the circadian activity of the intestinal microbiome through biological clock mechanisms (intestinal epithelial peristalsis, secretion) and dietary behaviors. Diurnal alterations of the intestinal microbiome in turn influence the rhythmic behavior of the host. A loss of circadian rhythm increases the susceptibility to viral and bacterial infections, leading to metabolic disorders as the intestinal microbiomes help to resist the invasion of pathogens through ecological competition (Thaiss et al., 2015). The recoverable properties of gut microbiome diversity observed in this study were in accordance with the MARS 500 trial (Turroni et al., 2017).

### Fusobacteria responding to the diurnal cycle variations

Repeated measurements and ANOVA analysis showed that the levels of *Fusobacteria* were significantly higher when MDC was performed (S8, S9 and S10) compared to EDC, indicating that the diurnal cycle caused gut microbiome variations at the phylum level. Fusobacteria are non-spore-forming, obligate anaerobic and gram-negative bacteria that frequently colonize the human oral cavity (Fan et al., 2018). It has been reported that boneC3G7 at the family level (phylum Fusobacteria) respond directly to diurnal circle variations, whilst the interactions between oral and intestinal microbiome may be enhanced (Derrien et al., 2010). Higher relative abundances of Fusobacteria were found in bronchial aspirates from patients with severe asthma (Millares et al., 2017). Furthermore, significantly increased levels were observed in the inflammatory bowel disease group (Santoru et al., 2018). Thus, further attention should be paid to explore the effects of increased Fusobacteria abundance when MDC is performed. Improper dietary control influences the gut microbiome leading to a loss of body weight (Maslowski & Mackay, 2011), and the psychological stress caused by limited space constraints and long-term isolation, also challenges the gut microbiome and health (Marchesi et al., 2016). During the Mars 500 trial, the structure of the bacterial flora also changed throughout the trial period. Specifically, the relative abundance of the anti-inflammatory bacterium Faecalibacterium significantly changed in the latter half of the year, reaching its lowest point after 1 year of entry (Turroni et al., 2017). Food provisions were identical for all crewmembers, but repeated ANOVA measurements of specific fecal microbiome at the phylum and genus level of the four crewmembers showed significant differences (Table 1), indicating that the individuals responded differently to the same dietary patterns. These individual responses may be due to differences in their original inherent gut microbiome and physiological state (Orland et al., 2019).

### Dietary and serum 25-hydroxyvitamin D variations

As shown in Fig. S4, decreases in BMI, energy intake and energy supply ratio by fat were common amongst the four crewmembers, which was in accordance with the dynamic variation of the Firmicutes: Bacteroidetes ratio (Fig. S7). The lower body weight and Firmicutes:Bacteroidetes (F:B) ratio during later experimental stages was related to energy restriction and psychological pressure (Bailey et al., 2011; Xu et al., 2016). Vitamin D_3_ is a fat-soluble vitamin partly derived from the dietary supply. It is mainly found in animal foods such as marine fish, animal liver, egg yolk and lean meat. Vitamin D_3_ can also be synthesized in a UV dependent manner (Ann, 2006). As the experiment was performed in an artificially enclosed environment, natural light was limited leading to decreased levels of vitamin D_3_ synthesis. After leaving the CELSS, the occupants were exposed to natural light and their diet was more diversified, boosting the body’s ability to synthesize vitamin D_3_ (Pasco et al., 2001). The levels of serum 25-hydroxyvitamin D of the four crewmembers increased at S5 compared to S4 which may have been due to the rise in inflammation inducing Fusobacteria (Bora et al., 2018). Consistent with these findings, Vitamin D_3_ levels of crewmembers have been reported to decrease during space-flight missions (Lane et al., 2013; Smith et al., 2005). Vitamin D_3_ supplements are therefore required to overcome this deficiency.

## Conclusions

A controlled ecological system environment provides an important model for future long-term manned space flight activities and life support for extraterrestrial planetary bases. During studies in the CELSS, diet and diurnal cycles underwent major changes, leading to variations of the gut microbiome. The dynamic changes of Firmicutes and Bacteroidetes were contrasting during the transitional period of the diurnal cycles. The gut microbiome of the crewmembers responded differently to identical dietary and environmental patterns both at the phylum and genus level. Both the unique environment of the CELSS, and MDC treatment caused significant differences of bacteria at the phylum and genus levels. Decreased BMI was correlated to decreased energy intake, which was in accordance with the increased F:B ratio during the later experimental stages. A consistent decrease in serum 25-hydroxyvitamin D was observed, most likely due to sunlight deprivation. The factors affecting the human gut microbiome diversity included the environment, diet, psychological stress and physical activity. Dynamic variations of the enterotypes for the four crewmembers as Bacteroides-Prevotella-Bacteroides-Prevotella during the isolated residence was a unique phenomenon under compound factors. Therefore, for long-term residence under a controlled ecological health system environment, it is necessary to study multi-factorial adjustment measures to regulate the gut microbiome, maintaining its diversity. As this study was limited by its small sample size, meaningful large-scale studies with close coordination of menu and environmental parameters should now be performed to verify our findings.

## Supplemental Information

10.7717/peerj.7762/supp-1 Supplemental Information 1 Relative abundance variation profiling of fecal microbiota at the order, family and species levels (top 20). Sequenced C0#S01 to C0#S15 refer to data from fecal samples for crewmember 0# 30 days before entering the CELSS, 15 days before entering the CELSS, and 2d, 15d, 30d, 45d, 60d, 75d, 90d, 105d, 120d, 135d, 150d, 165d and 175d after entering the CELSS. OC, period when crewmembers were out of the CELSS (before entry); IC, period when the crewmembers were in the CELSS.

10.7717/peerj.7762/supp-2 Supplemental Information 2 Core microbiome for each crewmembers at the phylum and genus levels (top 10). C01, C02, C03 and C04 refers to crewmember 01, 02, 03 and 04.

10.7717/peerj.7762/supp-3 Supplemental Information 3 Temporal variations of the top two phylum. S1 to S15 refers to fecal sampling time points: 15 days before entering the CELSS, and 2d, 15d, 30d, 45d, 60d, 75d, 90d, 105d, 120d, 135d, 150d, 165d and 175d after entering the CELSS.

10.7717/peerj.7762/supp-4 Supplemental Information 4 Temporal variation of the top two genus.

10.7717/peerj.7762/supp-5 Supplemental Information 5 Variation dynamics of alpha diversity (Richness and Evenness).

10.7717/peerj.7762/supp-6 Supplemental Information 6 Variations of serum 25-hydroxyvitamin D, α-tocopherol, retinol and folate concentrations during the experiment. Sequenced S01 to S09 refers to data from serum samples days before entry (S1), 2d in the CELSS (S2), 30d (S3), 60d (S4), 90d (S5), 120d (S6), 150d (S7), 175d (S8) and 30 days after exit from the CELSS (S9)

10.7717/peerj.7762/supp-7 Supplemental Information 7 Temporal variation dynamics of the Firmicutes:Bacteroidetes ratio.

10.7717/peerj.7762/supp-8 Supplemental Information 8 Statistics of the area and species of crops in the four modules of the system.

10.7717/peerj.7762/supp-9 Supplemental Information 9 Composition of 7-days scheduled recipes from day 1 to day 180.

10.7717/peerj.7762/supp-10 Supplemental Information 10 Sampling and grouping strategy for taxa analysis on the online statistical analysis platform Calypso Version 8.84.

10.7717/peerj.7762/supp-11 Supplemental Information 11 OTU table for taxa analysis on the online statistical analysis platform Calypso Version 8.84.

## Additional Information and Declarations

### Data Availability

The following information was supplied regarding data availability: The raw sequence data reads is available at NCBI SRA: PRJNA557020. The diet data is available as a Supplemental File.
