# Supplementary figures and images for: Simulated manned Mars exploration: effects of dietary and diurnal cycle variations on the gut microbiome of crew members in a controlled ecological life support system
Source: PeerJ. 2019 Sep 26;7:e7762. doi: 10.7717/peerj.7762 (PMC6766369; doi:10.7717/peerj.7762)

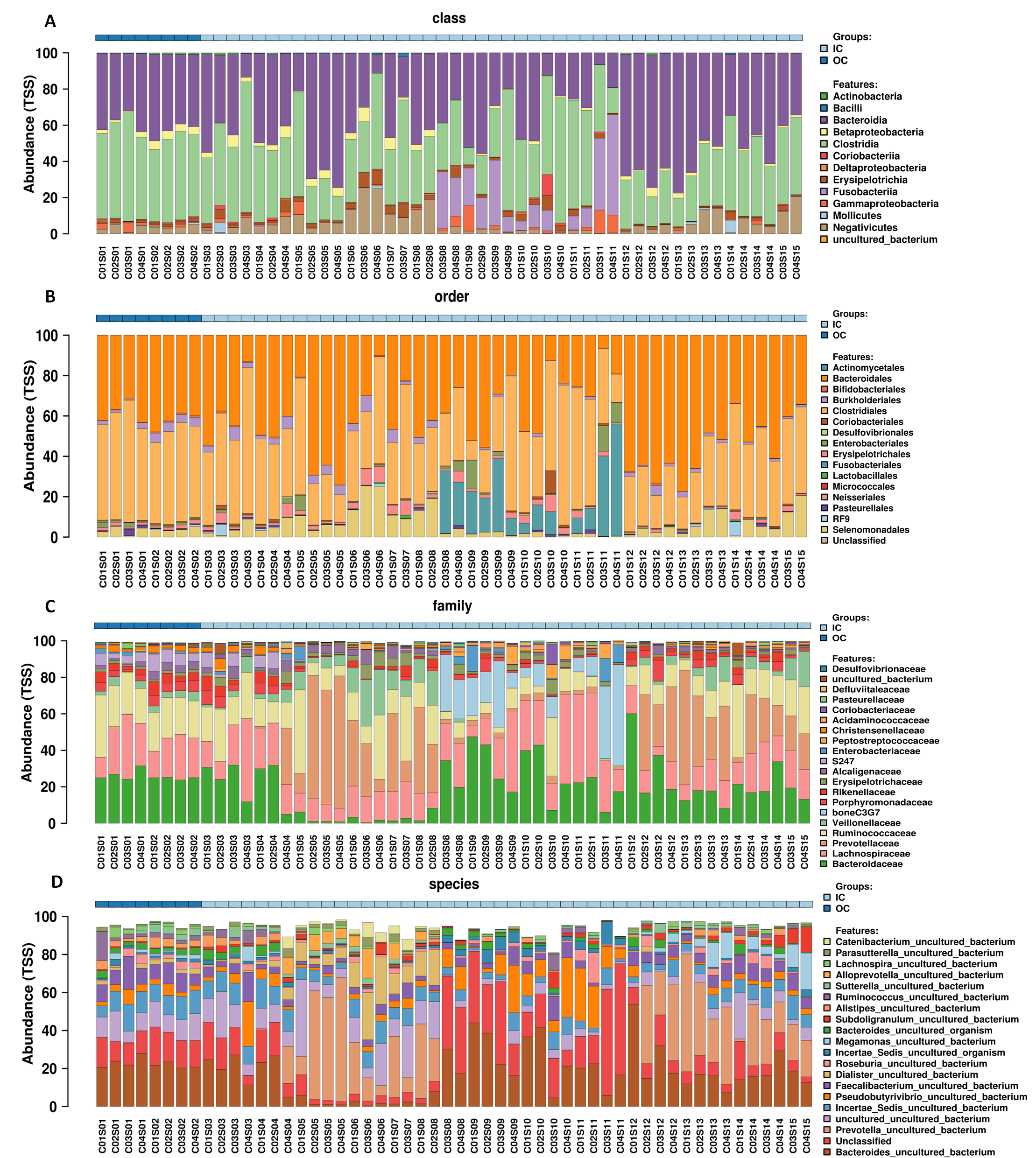

Supplement: Supplemental Information 1 — Sequenced C0#S01 to C0#S15 refer to data from fecal samples for crewmember 0# 30 days before entering the CELSS, 15 days before entering the CELSS, and 2d, 15d, 30d, 45d, 60d, 75d, 90d, 105d, 120d, 135d, 150d, 165d and 175d after entering the CELSS. OC, period when crewmembers were out of the CELSS (before entry); IC, period when the crewmembers were in the CELSS. [file peerj-07-7762-s001.png]

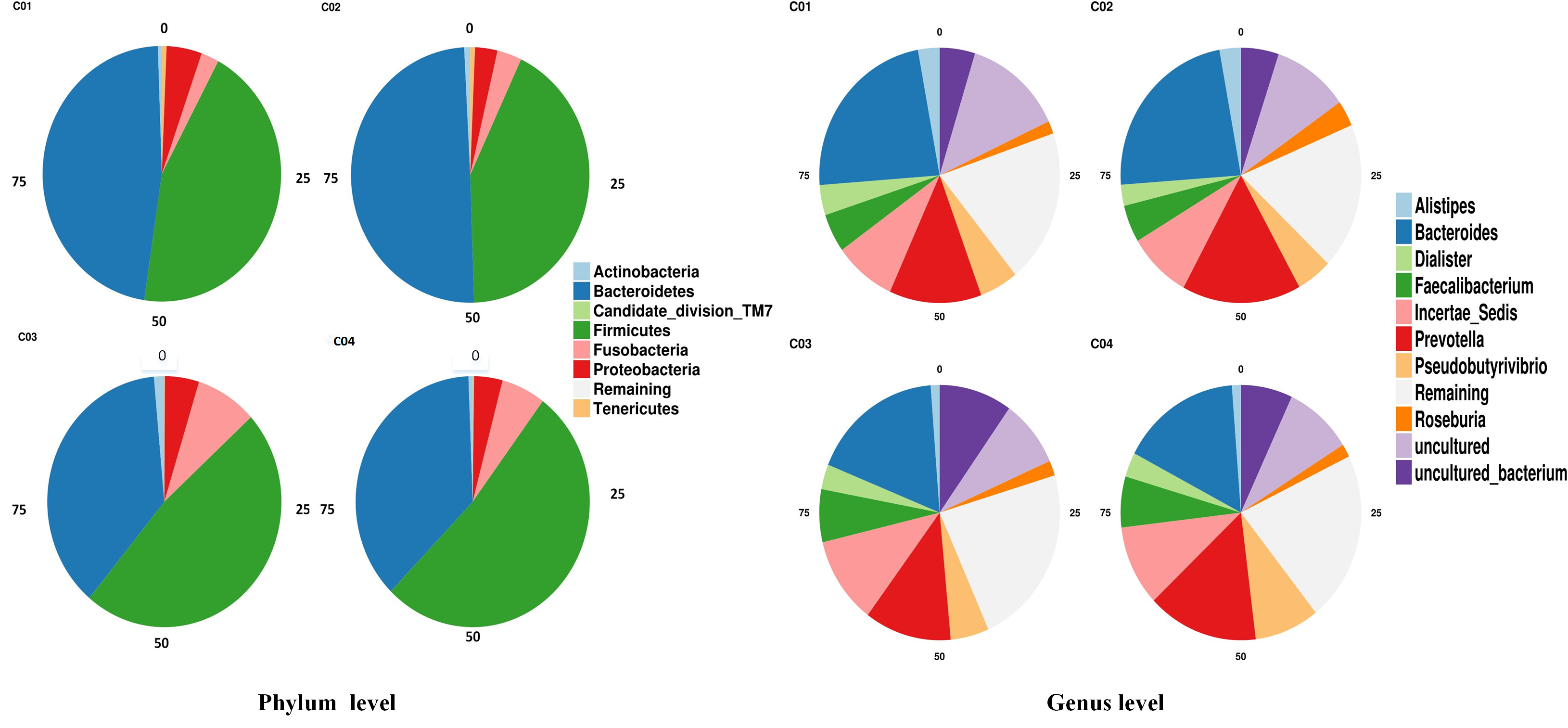

Supplement: Supplemental Information 2 — C01, C02, C03 and C04 refers to crewmember 01, 02, 03 and 04. [file peerj-07-7762-s002.png]

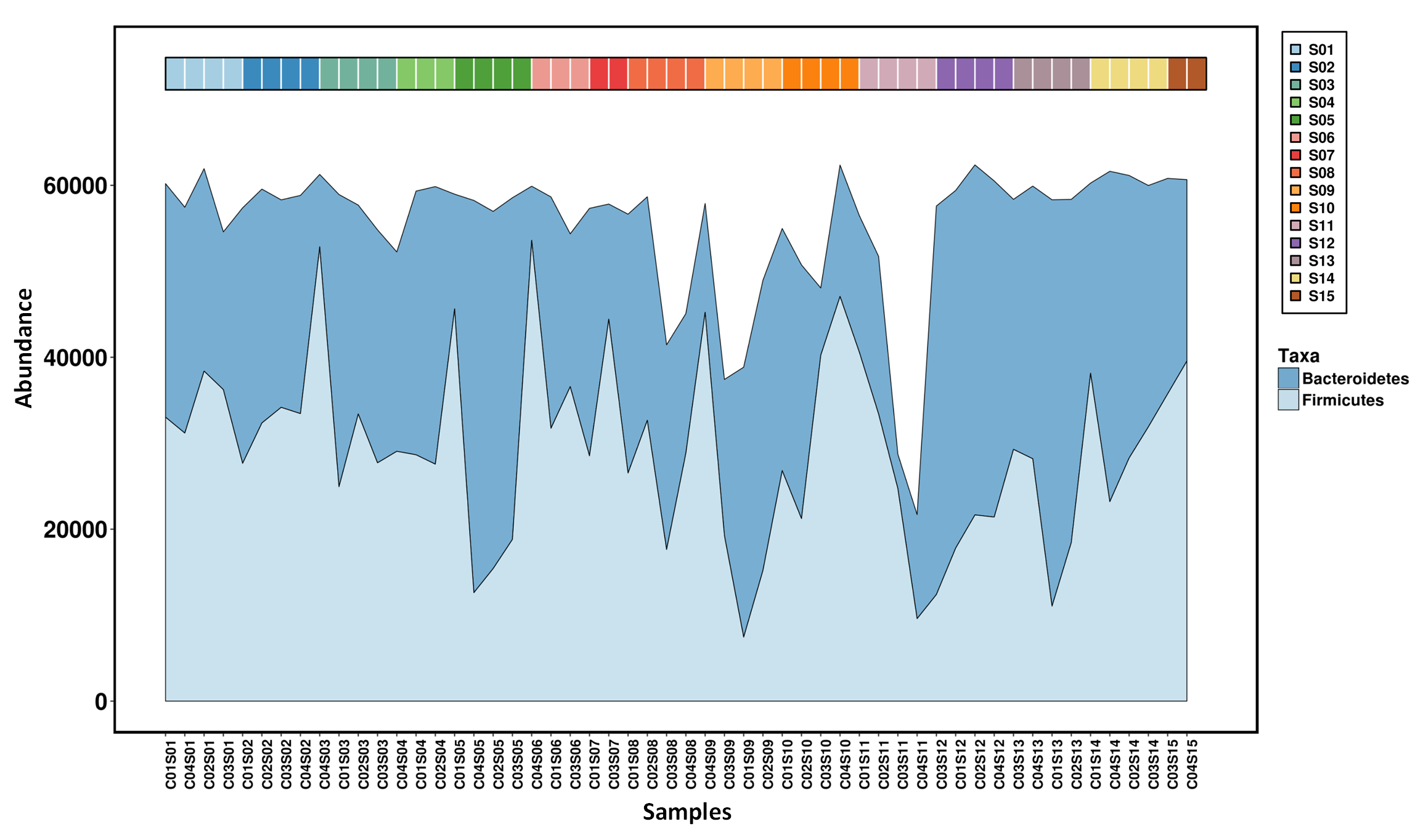

Supplement: Supplemental Information 3 — S1 to S15 refers to fecal sampling time points: 15 days before entering the CELSS, and 2d, 15d, 30d, 45d, 60d, 75d, 90d, 105d, 120d, 135d, 150d, 165d and 175d after entering the CELSS. [file peerj-07-7762-s003.png]

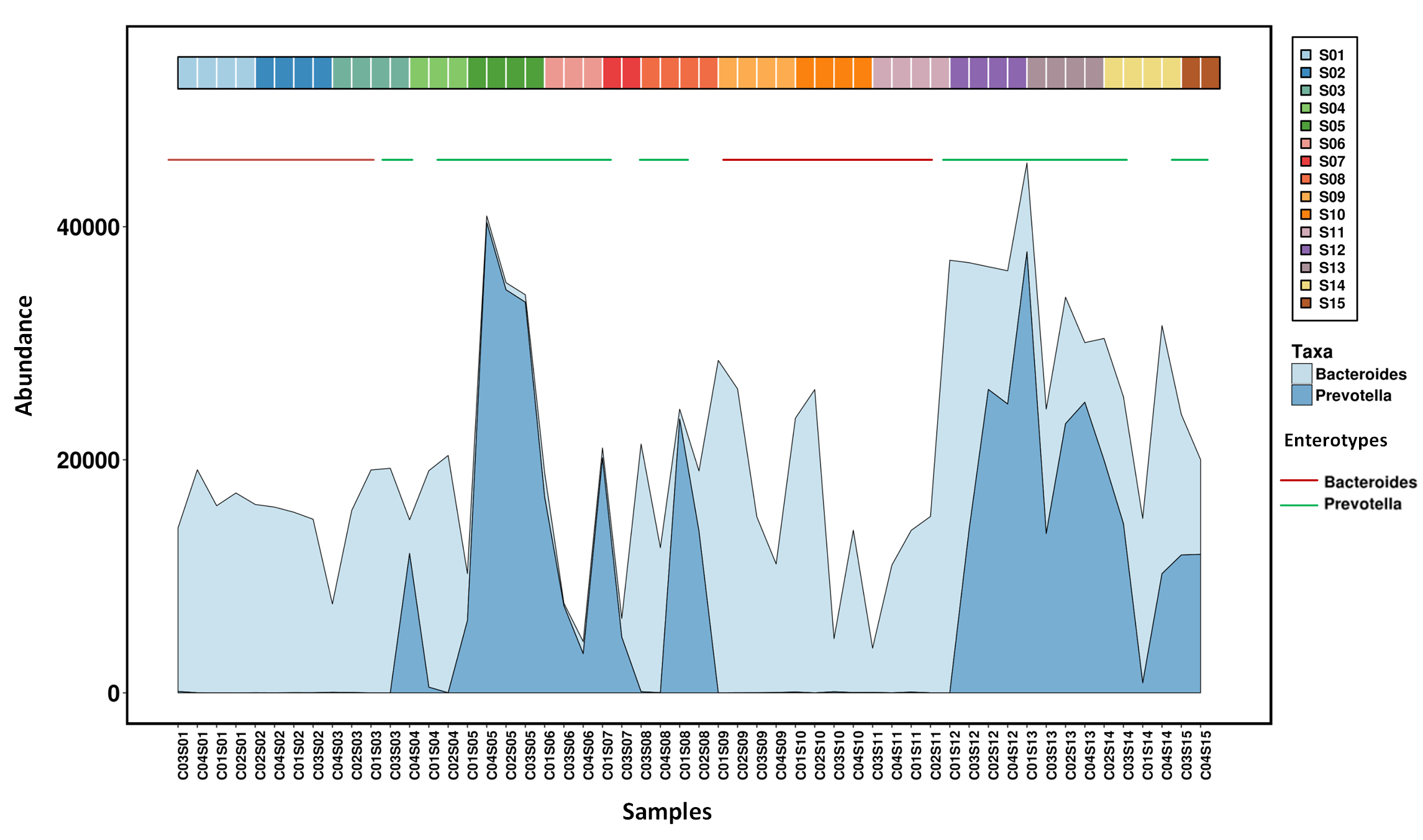

Supplement: Supplemental Information 4 [file peerj-07-7762-s004.png]

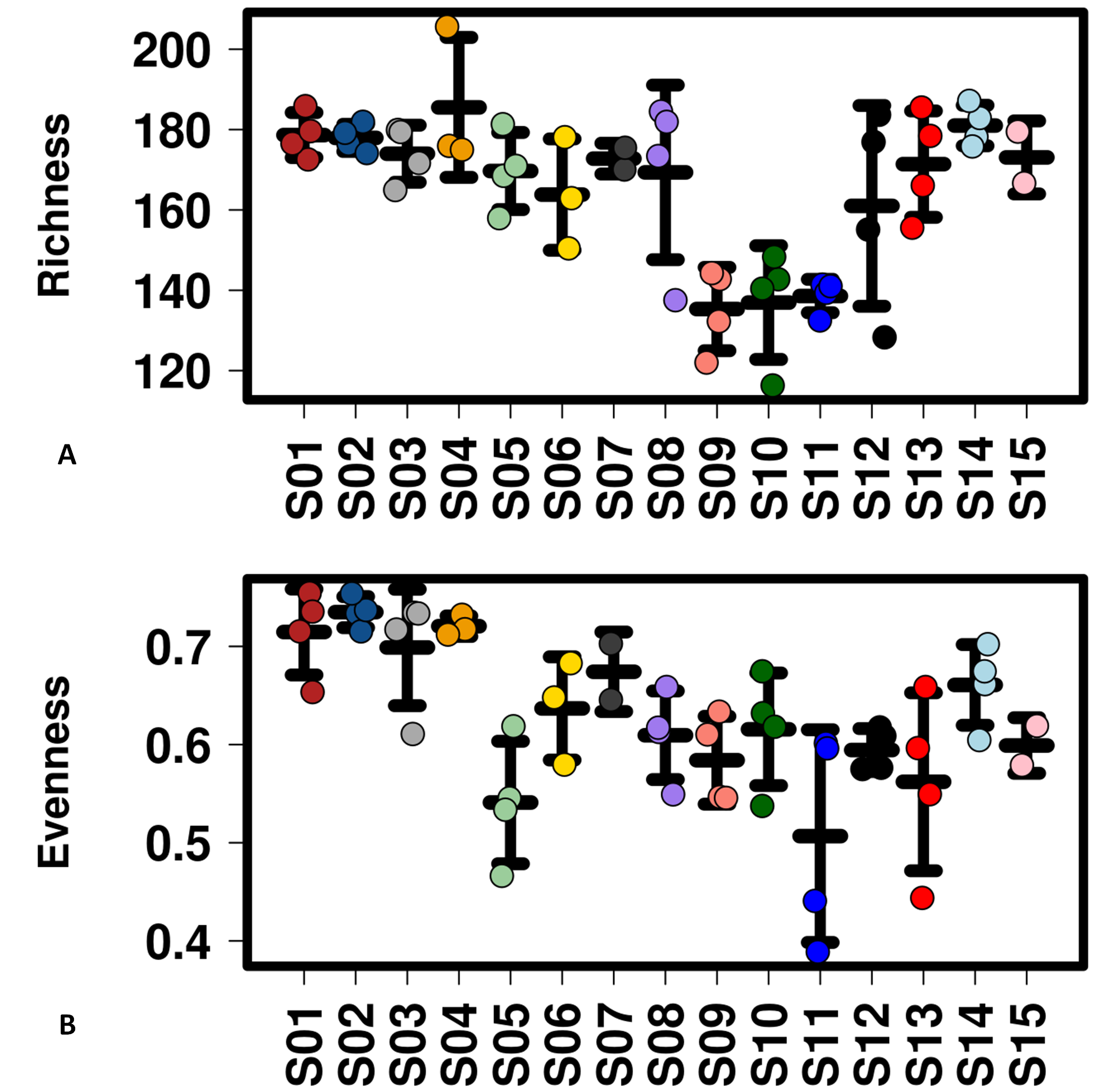

Supplement: Supplemental Information 5 [file peerj-07-7762-s005.png]

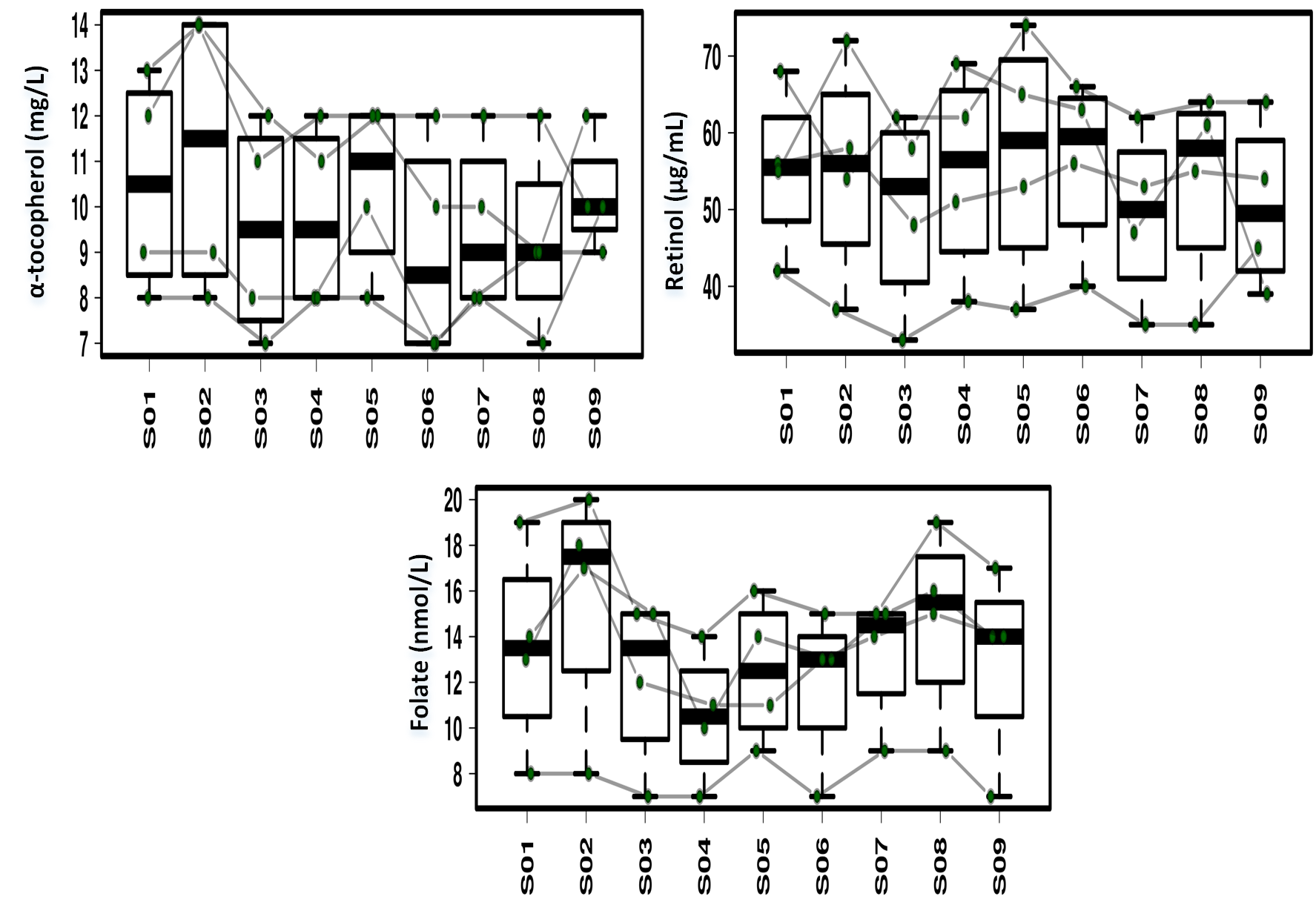

Supplement: Supplemental Information 6 — Sequenced S01 to S09 refers to data from serum samples days before entry (S1), 2d in the CELSS (S2), 30d (S3), 60d (S4), 90d (S5), 120d (S6), 150d (S7), 175d (S8) and 30 days after exit from the CELSS (S9) [file peerj-07-7762-s006.png]

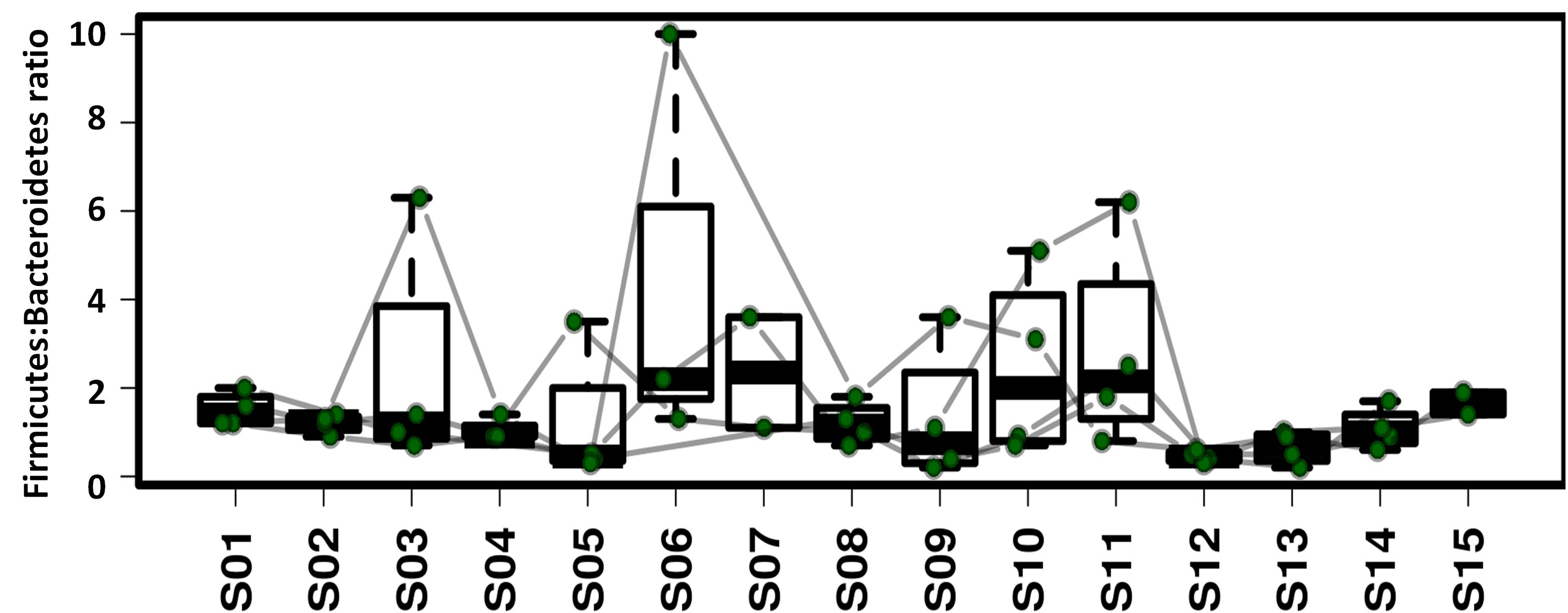

Supplement: Supplemental Information 7 [file peerj-07-7762-s007.png]
