# Supplementary material for: Simulated manned Mars exploration: effects of dietary and diurnal cycle variations on the gut microbiome of crew members in a controlled ecological life support system
Source: PeerJ. 2019 Sep 26;7:e7762. doi: 10.7717/peerj.7762 (PMC6766369; doi:10.7717/peerj.7762)
Supplement: Supplemental Information 8 [file peerj-07-7762-s008.docx]

**Table S1.** Statistics of the area and species of crops in the four modules of the system

| **Modules** | **Crops, vegetables and fruits** | **Area of crops (m^2^)** | **Subtotal** |
| --- | --- | --- | --- |
| Plant Module I | Sweet Potato | 13.86 | 63.84 |
|  | Peanut | 12.60 |  |
|  | Soy | 15.12 |  |
|  | Fruits And Vegetables* | 22.26 |  |
| Plant Module II | Wheat | 58.80 | 86.52 |
|  | Potato | 27.72 |  |
| Plant Module III | Wheat | 29.40 | 29.40 |
| Plant Module IV | Wheat | 7.24 | 12.85 |
|  | Fruits And Vegetables* | 5.61 |  |
| Summary | | 192.61 | |

*Fruits and vegetables: including edible amaranth, spinach, begonia, lactuca sativa l, lettuce, chinese parsley, chives, carrot, cabbage, celery, tomatoes, cucumber, bitter chrysanthemum, strawberry, green pepper, sweet potato leaf, pleurotus ostreatus, dendrobium, moringa, spirulina.
