# Supplementary material for: Simulated manned Mars exploration: effects of dietary and diurnal cycle variations on the gut microbiome of crew members in a controlled ecological life support system
Source: PeerJ. 2019 Sep 26;7:e7762. doi: 10.7717/peerj.7762 (PMC6766369; doi:10.7717/peerj.7762)
Supplement: Supplemental Information 9 [file peerj-07-7762-s009.docx]

**Table S2.** Composition of 7-days scheduled recipes from day 1 to day 180

**Table S2-1** Recipe No.1: Day1/8/15/22/29/36

| Meal | Classification | Dishes/item | Weight（g） | Calorie（kcal） |
| --- | --- | --- | --- | --- |
| Breakfast | Staple food | Bread | 175 | 529.1 |
|  | Staple food | Milk | 120 | 107.2 |
|  | Non-staple food (dishes) | Mixing vegetables | 80 | 86.6 |
|  | Non-staple food (dishes) | Sauce | 15 | 9.0 |
|  | Non-staple food (dishes) | Spiced corn and fried eggs | 45 | 69.3 |
|  | Others | Instant spirulina | 10 | 2.8 |
|  | Condiment | Condiment | 20 | 54.0 |
|  | Subtotal |  | 465 | 858 |
| Lunch | Staple food | Steamed rice | 200 | 235.8 |
|  | Non-staple food (dishes) | Vegetable salad | 180 | 115.3 |
|  | Non-staple food (dishes) | Pickles / sauce | 15 | 9.0 |
|  | Non-staple food (dishes) | Vegetable soup | 100 | 63.0 |
|  | Non-staple food (dishes) | Barbecued chicken | 40 | 78.4 |
|  | Non-staple food (dishes) | Cabbage stewed rabbit meat | 80 | 134.9 |
|  | Condiment | Condiment | 10 | / |
|  | Subtotal |  | 625 | 636.4 |
| Supper | Staple food | Noodles | 200 | 470.3 |
|  | Non-staple food (dishes) | Carrot stewed duck | 80 | 113.9 |
|  | Non-staple food (dishes) | Microwave dishes | 180 | 194.9 |
|  | Non-staple food (dishes) | Sauced beef | 40 | 62.0 |
|  | Non-staple food (dishes) | Pickles / sauce | 15 | 9.0 |
|  | Non-staple food (dishes) | Vegetable soup | 100 | 63.0 |
|  | Condiment | Condiment | 10 | / |
|  | Subtotal |  | 625 | 913.1 |
| Refreshments | Ready to eat | Fruit | 100 | 34.2 |
|  |  | Biscuits | 40 | 161.6 |
|  |  | Bean strip | 20 | 53.9 |
|  |  | Chocolate | 14 | 72.7 |
|  | Sum |  | 1889 | 2730 |

**Table S2-2** Recipe No.2: Day2/9/16/23/30/37

| Meal | Classification | Dishes/item | Weight（g） | Calorie（kcal） |
| --- | --- | --- | --- | --- |
| Breakfast | Staple food | Flour soup | 200.0 | 253.1 |
|  | Non-staple food (dishes) | Peanut | 10.0 | 59.8 |
|  | Non-staple food (dishes) | Mixing vegetables | 80 | 86.6 |
|  | Non-staple food (dishes) | Pickles / sauce | 15.0 | 9.0 |
|  | Non-staple food (dishes) | Spiced quail eggs | 35.0 | 96.8 |
|  | Others | Instant spirulina | 10.0 | 2.8 |
|  | Condiment | Condiment | 10.0 | 27.0 |
|  | Subtotal |  | 360 | 535.1 |
| Lunch | Staple food | Noodles | 200.0 | 470.3 |
|  | Non-staple food (dishes) | Vegetable salad | 180.0 | 115.3 |
|  | Non-staple food (dishes) | Pickles / sauce | 15.0 | 9.0 |
|  | Non-staple food (dishes) | Vegetable soup | 100.0 | 63.0 |
|  | Non-staple food (dishes) | Black Pepper Beef | 80.0 | 134.2 |
|  | Non-staple food (dishes) | Spicy tuna | 40.0 | 59.2 |
|  | Condiment | Condiment | 10.0 | / |
|  | Subtotal |  | 625 | 851 |
| Supper | Staple food | Dough piece | 200.0 | 470.3 |
|  | Non-staple food (dishes) | Microwave dishes | 180.0 | 194.9 |
|  | Non-staple food (dishes) | Pickles / sauce | 15.0 | 9.0 |
|  | Non-staple food (dishes) | Vegetable soup | 100.0 | 63.0 |
|  | Non-staple food (dishes) | Shrimp egg | 80.0 | 118.2 |
|  | Non-staple food (dishes) | Spicy chicken wings | 40.0 | 70.4 |
|  | Condiment | Condiment | 10.0 | / |
|  | Subtotal |  | 625 | 925.8 |
| Refreshments | Ready to eat | Fruit | 100.0 | 34.2 |
|  |  | Biscuits | 60.0 | 242.5 |
|  |  | Yogurt | 150 | 159.2 |
|  |  | Chocolate | 14 | 72.7 |
|  | Sum |  | 1934 | 2821 |

**Table S2-3** Recipe No.3: Day3/10/17/24/31/38

| Meal | Classification | Dishes/item | Weight（g） | Calorie（kcal） |
| --- | --- | --- | --- | --- |
| Breakfast | Staple food | Bread | 175 | 529.1 |
|  | Staple food | Milk | 120 | 107.2 |
|  | Non-staple food (dishes) | Mixing vegetables | 80 | 86.6 |
|  | Non-staple food (dishes) | Sauce | 15 | 9.0 |
|  | Non-staple food (dishes) | Spiced corn and fried eggs | 45 | 69.3 |
|  | Others | Instant spirulina | 10 | 2.8 |
|  | Condiment | Condiment | 10 | 27.0 |
|  | Subtotal |  | 455 | 831 |
| Lunch | Staple food | Noodles | 200 | 470.3 |
|  | Non-staple food (dishes) | Vegetable salad | 180 | 115.3 |
|  | Non-staple food (dishes) | Pickles / sauce | 15 | 9.0 |
|  | Non-staple food (dishes) | Vegetable soup | 100 | 63.0 |
|  | Non-staple food (dishes) | Milky chicken corn | 80 | 94.9 |
|  | Non-staple food (dishes) | Pork sausage | 40 | 104.7 |
|  | Condiment | Condiment | 10 | / |
|  | Subtotal |  | 625 | 857.2 |
| Supper | Staple food | Steamed rice | 200 | 233.4 |
|  | Non-staple food (dishes) | Microwave dishes | 180 | 194.9 |
|  | Non-staple food (dishes) | Pickles / sauce | 15 | 9.0 |
|  | Non-staple food (dishes) | Vegetable soup | 100 | 63.0 |
|  | Non-staple food (dishes) | Steamed tendon | 80 | 160.7 |
|  | Non-staple food (dishes) | Cardamom | 40 | 83.6 |
|  | Condiment | Condiment | 10 | / |
|  | Subtotal |  | 625 | 744.6 |
| Refreshments | Ready to eat | Fruit | 100 | 34.2 |
|  |  | Biscuits | 40 | 161.6 |
|  |  | Bean strip | 20 | 53.9 |
|  |  | Chocolate | 14 | 72.7 |
|  | Sum |  | 1879 | 2755 |

**Table S2-4** Recipe No.4: Day4/11/18/25/32/39

| Meal | Classification | Dishes/item | Weight（g） | Calorie（kcal） |
| --- | --- | --- | --- | --- |
| Breakfast | Staple food | Flour soup | 200 | 253.1 |
|  | Non-staple food (dishes) | Peanut | 10 | 59.8 |
|  | Non-staple food (dishes) | Mixing vegetables | 80 | 86.6 |
|  | Non-staple food (dishes) | Pickles / sauce | 15 | 9.0 |
|  | Non-staple food (dishes) | Spiced quail eggs | 35 | 96.8 |
|  | Others | Instant spirulina | 10 | 2.8 |
|  | Condiment | Condiment | 10 | 27.0 |
|  | Subtotal |  | 360 | 535.1 |
| Lunch | Staple food | Noodles | 200 | 470.3 |
|  | Non-staple food (dishes) | Vegetable salad | 180 | 115.3 |
|  | Non-staple food (dishes) | Pickles / sauce | 15 | 9.0 |
|  | Non-staple food (dishes) | Vegetable soup | 100 | 63.0 |
|  | Non-staple food (dishes) | Spicy lamb | 80 | 138.7 |
|  | Non-staple food (dishes) | Fragrant chicken | 40 | 60.7 |
|  | Condiment | Condiment | 10 | / |
|  | Subtotal |  | 625 | 857 |
| Supper | Staple food | Steamed cake | 150 | 393.5 |
|  | Staple food | Millet porridge | 200 | 91.0 |
|  | Non-staple food (dishes) | Microwave dishes | 180 | 194.9 |
|  | Non-staple food (dishes) | Pickles / sauce | 15 | 9.0 |
|  | Non-staple food (dishes) | Edible tree fungusbeef | 80 | 86.6 |
|  | Non-staple food (dishes) | Fragrant pork ear | 40 | 88.4 |
|  | Condiment | Condiment | 10 | / |
|  | Subtotal |  | 675 | 863.4 |
| Refreshments | Ready to eat | Fruit | 100 | 34.2 |
|  |  | Biscuits | 60 | 242.5 |
|  |  | Yogurt | 150 | 159.2 |
|  |  | Chocolate | 14 | 72.7 |
|  | Sum |  | 1984 | 2764 |

**Table S2-5** Recipe No.5: Day5/12/19/26/33/40

| Meal | Classification | Dishes/item | Weight（g） | Calorie（kcal） |
| --- | --- | --- | --- | --- |
| Breakfast | Staple food | Bread | 175 | 529.1 |
|  | Staple food | Milk | 120 | 107.2 |
|  | Non-staple food (dishes) | Mixing vegetables | 80 | 86.6 |
|  | Non-staple food (dishes) | Sauce | 15 | 9.0 |
|  | Non-staple food (dishes) | Spiced corn and fried eggs | 45 | 69.3 |
|  | Others | Instant spirulina | 10 | 2.8 |
|  | Condiment | Condiment | 10 | 27.0 |
|  | Subtotal |  | 455 | 831 |
| Lunch | Staple food | Noodles | 200 | 470.3 |
|  | Non-staple food (dishes) | Vegetable salad | 180 | 115.3 |
|  | Non-staple food (dishes) | Pickles / sauce | 15 | 9.0 |
|  | Non-staple food (dishes) | Vegetable soup | 100 | 63.0 |
|  | Non-staple food (dishes) | Bazhen chicken | 80 | 117.7 |
|  | Non-staple food (dishes) | Pingyaobeef | 40 | 61.2 |
|  | Condiment | Condiment | 10 | / |
|  | Subtotal |  | 625 | 836.5 |
| Supper | Staple food | Steamed bread | 150 | 338.9 |
|  | Staple food | Rice porridge | 100 | 46.7 |
|  | Non-staple food (dishes) | Microwave dishes | 180 | 194.9 |
|  | Non-staple food (dishes) | Pickles / sauce | 15 | 9.0 |
|  | Non-staple food (dishes) | Muxurou | 80 | 122.0 |
|  | Non-staple food (dishes) | Spicy goose | 40 | 67.4 |
|  | Condiment | Condiment | 10 | / |
|  | Subtotal |  | 575 | 778.9 |
| Refreshments | Ready to eat | Fruit | 100 | 34.2 |
|  |  | Biscuits | 40 | 161.6 |
|  |  | Bean strip | 20 | 53.9 |
|  |  | Chocolate | 14 | 72.7 |
|  | Sum |  | 1829 | 2769 |

**Table S2-6** Recipe No.6: Day6/13/20/27/34

| Meal | Classification | Dishes/item | Weight（g） | Calorie（kcal） |
| --- | --- | --- | --- | --- |
| Breakfast | Staple food | Flour soup | 200 | 253.1 |
|  | Non-staple food (dishes) | Peanut | 10 | 59.8 |
|  | Non-staple food (dishes) | Mixing vegetables | 80 | 86.6 |
|  | Non-staple food (dishes) | Pickles / sauce | 15 | 9.0 |
|  | Others | Instant spirulina | 10 | 2.8 |
|  | Non-staple food (dishes) | Spiced quail eggs | 35 | 96.8 |
|  | Condiment | Condiment | 10 | 27.0 |
|  | Subtotal |  | 360 | 535.1 |
| Lunch | Staple food | Noodles | 200 | 470.3 |
|  | Non-staple food (dishes) | Vegetable salad | 180 | 115.3 |
|  | Non-staple food (dishes) | Pickles / sauce | 15 | 9.0 |
|  | Non-staple food (dishes) | Vegetable soup | 100 | 63.0 |
|  | Non-staple food (dishes) | Pork chops | 80 | 177.4 |
|  | Condiment | Condiment | 10 | / |
|  | Subtotal |  | 585 | 835 |
| Supper | Staple food | Steamed cake | 150 | 393.5 |
|  |  | Millet porridge | 200 | 91.0 |
|  | Non-staple food (dishes) | Microwave dishes | 180 | 194.9 |
|  | Non-staple food (dishes) | Pickles / sauce | 15 | 9.0 |
|  | Non-staple food (dishes) | Spicy duck | 80 | 201.8 |
|  | Condiment | Condiment | 10 | / |
|  | Subtotal |  | 635 | 890.2 |
| Refreshments | Ready to eat | Fruit | 100 | 34.2 |
|  |  | Biscuits | 60 | 242.5 |
|  |  | Yogurt | 150 | 159.2 |
|  |  | Chocolate | 14 | 72.7 |
|  | Sum |  | 1904 | 2769 |

**Table S2-7** Recipe No.7: Day7/14/21/28/35

| Meal | Classification | Dishes/item | Weight（g） | Calorie（kcal） |
| --- | --- | --- | --- | --- |
| Breakfast | Staple food | Bread | 175 | 529.1 |
|  | Staple food | Milk | 120 | 107.2 |
|  | Non-staple food (dishes) | Mixing vegetables | 80 | 86.6 |
|  | Non-staple food (dishes) | Sauce | 15 | 9.0 |
|  | Non-staple food (dishes) | Spiced corn and fried eggs | 45 | 69.3 |
|  | Others | Instant spirulina | 10 | 2.8 |
|  | Condiment | Condiment | 10 | 27.0 |
|  | Subtotal |  | 455 | 831 |
| Lunch | Staple food | Noodles | 200 | 470.3 |
|  | Non-staple food (dishes) | Vegetable salad | 180 | 115.3 |
|  | Non-staple food (dishes) | Pickles / sauce | 15 | 9.0 |
|  | Non-staple food (dishes) | Vegetable soup | 100 | 63.0 |
|  | Non-staple food (dishes) | Roast chicken leg | 80 | 176.5 |
|  | Non-staple food (dishes) | Spicy pork | 40 | 79.1 |
|  | Condiment | Condiment | 10 | / |
|  | Subtotal |  | 625 | 913.2 |
| Supper | Staple food | Dumplings | 200 | 535.2 |
|  | Non-staple food (dishes) | Mixing vegetables | 80 | 86.6 |
|  | Non-staple food (dishes) | Pickles / sauce | 15 | 9.0 |
|  | Non-staple food (dishes) | Spicy pork | 80 | 0.0 |
|  | Non-staple food (dishes) | Black pepper duck | 40 | 115.4 |
|  | Condiment | Condiment | 10 | / |
|  | Subtotal |  | 425 | 746.2 |
| Refreshments | Ready to eat | Fruit | 100 | 34.2 |
|  |  | Biscuits | 40 | 161.6 |
|  |  | Bean strip | 20 | 53.9 |
|  |  |  |  |  |
|  | Sum |  | 1665 | 2740 |

**Table S2-8** Recipe No.8: Day41/48/55

| Meal | Classification | Dishes/item | Weight（g） | Calorie（kcal） |
| --- | --- | --- | --- | --- |
| Breakfast | Staple food | Bread | 175 | 529.1 |
|  | Staple food | Milk | 120 | 107.2 |
|  | Non-staple food (dishes) | Mixing vegetables | 80 | 86.6 |
|  | Non-staple food (dishes) | Sauce | 15 | 9.0 |
|  | Non-staple food (dishes) | Spiced corn and fried eggs | 45 | 69.3 |
|  | Others | Instant spirulina | 10 | 2.8 |
|  | Condiment | Condiment | 20 | 54.0 |
|  | Subtotal |  | 465 | 858 |
| Lunch | Staple food | Steamed rice | 200 | 235.8 |
|  | Non-staple food (dishes) | Vegetable salad | 180 | 115.3 |
|  | Non-staple food (dishes) | Pickles / sauce | 15 | 9.0 |
|  | Non-staple food (dishes) | Vegetable soup | 100 | 63.0 |
|  | Non-staple food (dishes) | Cabbage stewed rabbit meat | 80 | 134.9 |
|  | Non-staple food (dishes) | Barbecued chicken | 40 | 78.4 |
|  | Condiment | Condiment | 10 | / |
|  | Subtotal |  | 625 | 636.4 |
| Supper | Staple food | Noodles | 200 | 470.3 |
|  | Non-staple food (dishes) | Spicy potato strip | 200 | 283.2 |
|  | Non-staple food (dishes) | Pickles / sauce | 15 | 9.0 |
|  | Non-staple food (dishes) | Vegetable soup | 100 | 63.0 |
|  | Non-staple food (dishes) | Carrot duck | 80 | 113.9 |
|  | Non-staple food (dishes) | Saucebeef | 40 | 62.0 |
|  | Condiment | Condiment | 10 | / |
|  | Subtotal |  | 645 | 1001.4 |
| Refreshments | Ready to eat | Fruit | 100 | 34.2 |
|  |  | Bean strip | 20 | 53.9 |
|  |  | Chocolate | 14 | 72.7 |
|  | Sum |  | 1869 | 2657 |

**Table S2-9** Recipe No.9: Day42/49/56

| Meal | Classification | Dishes/item | Weight（g） | Calorie（kcal） |
| --- | --- | --- | --- | --- |
| Breakfast | Staple food | Flour soup | 200 | 253.1 |
|  | Non-staple food (dishes) | Peanut | 10 | 59.8 |
|  | Non-staple food (dishes) | Mixing vegetables | 80 | 86.6 |
|  | Non-staple food (dishes) | Pickles / sauce | 15 | 9.0 |
|  | Non-staple food (dishes) | Spiced quail eggs | 35 | 96.8 |
|  | Others | Instant spirulina | 10 | 2.8 |
|  | Condiment | Condiment | 10 | 27.0 |
|  | Subtotal |  | 360 | 535.1 |
| Lunch | Staple food | Noodles | 200 | 470.3 |
|  | Non-staple food (dishes) | Vegetable salad | 180 | 115.3 |
|  | Non-staple food (dishes) | Pickles / sauce | 15 | 9.0 |
|  | Non-staple food (dishes) | Vegetable soup | 100 | 63.0 |
|  | Non-staple food (dishes) | Black Pepper Beef | 80 | 134.2 |
|  | Non-staple food (dishes) | Spicy tuna | 40 | 59.2 |
|  | Condiment | Condiment | 10 | / |
|  | Subtotal |  | 625 | 851 |
| Supper | Staple food | Dough piece | 200 | 470.3 |
|  | Non-staple food (dishes) | Microwave dishes | 180 | 194.9 |
|  | Non-staple food (dishes) | Pickles / sauce | 15 | 9.0 |
|  | Non-staple food (dishes) | Vegetable soup | 100 | 63.0 |
|  | Non-staple food (dishes) | Shrimp egg | 80 | 118.2 |
|  | Non-staple food (dishes) | Spicy chicken wings | 40 | 70.4 |
|  | Condiment | Condiment | 10 | / |
|  | Subtotal |  | 625 | 925.8 |
| Refreshments | Ready to eat | Fruit | 100 | 34.2 |
|  |  | Steamed potatoes | 100 | 109.6 |
|  |  | Yogurt | 150 | 159.2 |
|  |  | Chocolate | 14 | 72.7 |
|  | Sum |  | 1974 | 2688 |

**Table S2-10** Recipe No.10: Day43/50/57

| Meal | Classification | Dishes/item | Weight（g） | Calorie（kcal） |
| --- | --- | --- | --- | --- |
| Breakfast | Staple food | Rice porridge | 200 | 91.0 |
|  | Staple food | Steamed sweet potato | 100 | 108.2 |
|  | Non-staple food (dishes) | Mixing vegetables | 80 | 86.6 |
|  | Non-staple food (dishes) | Sauce | 15 | 9.0 |
|  | Non-staple food (dishes) | Spiced corn and fried eggs | 45 | 69.3 |
|  | Others | Instant spirulina | 10 | 2.8 |
|  | Condiment | Condiment | 10 | 27.0 |
|  | Subtotal |  | 460 | 393.9 |
| Lunch | Staple food | Noodles | 200 | 470.3 |
|  | Non-staple food (dishes) | Spicy potato strip | 200 | 283.2 |
|  | Non-staple food (dishes) | Pickles / sauce | 15 | 9.0 |
|  | Non-staple food (dishes) | Vegetable soup | 100 | 63.0 |
|  | Non-staple food (dishes) | Milky chicken corn | 80 | 94.9 |
|  | Non-staple food (dishes) | Pork sausage | 40 | 104.7 |
|  | Condiment | Condiment | 10 | / |
|  | Subtotal |  | 645 | 1025.1 |
| Supper | Staple food | Noodles | 200 | 470.3 |
|  | Non-staple food (dishes) | Microwave dishes | 180 | 194.9 |
|  | Non-staple food (dishes) | Pickles / sauce | 15 | 9.0 |
|  | Non-staple food (dishes) | Vegetable soup | 100 | 63.0 |
|  | Non-staple food (dishes) | Steamed tendon | 80 | 160.7 |
|  | Non-staple food (dishes) | Cardamom | 40 | 83.6 |
|  | Condiment | Condiment | 10 | / |
|  | Subtotal |  | 625 | 981.5 |
| Refreshments | Ready to eat | Fruit | 100 | 34.2 |
|  |  | Yogurt | 150 | 159.2 |
|  |  | Bean strip | 20 | 53.9 |
|  |  | Chocolate | 14 | 72.7 |
|  | Sum |  | 2014 | 2721 |

**Table S2-11** Recipe No.11: Day44/51/58

| Meal | Classification | Dishes/item | Weight（g） | Calorie（kcal） |
| --- | --- | --- | --- | --- |
| Breakfast | Staple food | Bread | 175 | 529.1 |
|  | Non-staple food (dishes) | Milk | 120 | 107.2 |
|  | Non-staple food (dishes) | Mixing vegetables | 80 | 86.6 |
|  | Non-staple food (dishes) | Pickles / sauce | 15 | 9.0 |
|  | Non-staple food (dishes) | Spiced quail eggs | 35 | 96.8 |
|  | Others | Instant spirulina | 10 | 2.8 |
|  | Condiment | Condiment | 10 | 27.0 |
|  | Subtotal |  | 445 | 858.5 |
| Lunch | Staple food | Noodles | 200 | 470.3 |
|  | Non-staple food (dishes) | Vegetable salad | 180 | 115.3 |
|  | Non-staple food (dishes) | Pickles / sauce | 15 | 9.0 |
|  | Non-staple food (dishes) | Vegetable soup | 100 | 63.0 |
|  | Non-staple food (dishes) | Spicy lamb | 80 | 138.7 |
|  | Non-staple food (dishes) | Fragrant chicken | 40 | 60.7 |
|  | Condiment | Condiment | 10 | / |
|  | Subtotal |  | 625 | 857 |
| Supper | Staple food | Steamed cake | 150 | 393.5 |
|  | Staple food | Millet porridge | 200 | 91.0 |
|  | Non-staple food (dishes) | Microwave dishes | 180 | 194.9 |
|  | Non-staple food (dishes) | Pickles / sauce | 15 | 9.0 |
|  | Non-staple food (dishes) | Edible tree fungusbeef | 80 | 86.6 |
|  | Non-staple food (dishes) | Fragrant pork ear | 40 | 88.4 |
|  | Condiment | Condiment | 10 | / |
|  | Subtotal |  | 675 | 863.4 |
| Refreshments | Ready to eat | Fruit | 100 | 34.2 |
|  |  | Steamed potatoes | 100 | 109.6 |
|  |  | Chocolate | 14 | 72.7 |
|  | Sum |  | 1959 | 2795 |

**Table S2-12** Recipe No.12: Day45/52/59

| Meal | Classification | Dishes/item | Weight（g） | Calorie（kcal） |
| --- | --- | --- | --- | --- |
| Breakfast | Staple food | Bread | 175 | 529.1 |
|  | Staple food | Milk | 120 | 107.2 |
|  | Non-staple food (dishes) | Spiced corn and fried eggs | 45 | 69.3 |
|  | Non-staple food (dishes) | Sauce | 15 | 9.0 |
|  | Non-staple food (dishes) | Mixing vegetables | 80 | 86.6 |
|  | Others | Instant spirulina | 10 | 2.8 |
|  | Condiment | Condiment | 10 | 27.0 |
|  | Subtotal |  | 455 | 831 |
| Lunch | Staple food | Noodles | 200 | 470.3 |
|  | Non-staple food (dishes) | Vegetable salad | 180 | 115.3 |
|  | Non-staple food (dishes) | Pickles / sauce | 15 | 9.0 |
|  | Non-staple food (dishes) | Vegetable soup | 100 | 63.0 |
|  | Non-staple food (dishes) | Bazhen chicken | 80 | 117.7 |
|  | Non-staple food (dishes) | Pingyaobeef | 40 | 61.2 |
|  | Condiment | Condiment | 10 | / |
|  | Subtotal |  | 625 | 836.5 |
| Supper | Staple food | Steamed bread | 150 | 338.9 |
|  | Staple food | Rice porridge | 100 | 46.7 |
|  | Non-staple food (dishes) | Spicy potato strip | 200 | 283.2 |
|  | Non-staple food (dishes) | Pickles / sauce | 15 | 9.0 |
|  | Non-staple food (dishes) | Muxurou | 80 | 122.0 |
|  | Non-staple food (dishes) | Spicy goose | 40 | 67.4 |
|  | Condiment | Condiment | 10 | / |
|  | Subtotal |  | 595 | 867.2 |
| Refreshments | Ready to eat | Fruit | 100 | 34.2 |
|  |  | Steamed sweet potato | 100 | 108.2 |
|  |  | Bean strip | 20 | 53.9 |
|  |  | Chocolate | 14 | 72.7 |
|  | Sum |  | 1909 | 2804 |

**Table S2-13** Recipe No.13: Day46/53/60

| Meal | Classification | Dishes/item | Weight（g） | Calorie（kcal） |
| --- | --- | --- | --- | --- |
| Breakfast | Staple food | Flour soup | 200 | 253.1 |
|  | Non-staple food (dishes) | Peanut | 10 | 59.8 |
|  | Non-staple food (dishes) | Mixing vegetables | 80 | 86.6 |
|  | Non-staple food (dishes) | Pickles / sauce | 15 | 9.0 |
|  | Non-staple food (dishes) | Spiced quail eggs | 35 | 96.8 |
|  | Others | Instant spirulina | 10 | 2.8 |
|  | Condiment | Condiment | 10 | 27.0 |
|  | Subtotal |  | 360 | 535.1 |
| Lunch | Staple food | Noodles | 200 | 470.3 |
|  | Non-staple food (dishes) | Vegetable salad | 180 | 115.3 |
|  | Non-staple food (dishes) | Pickles / sauce | 15 | 9.0 |
|  | Non-staple food (dishes) | Vegetable soup | 100 | 63.0 |
|  | Non-staple food (dishes) | Pork chops | 80 | 177.4 |
|  | Condiment | Condiment | 10 | / |
|  | Subtotal |  | 585 | 835 |
| Supper | Staple food | Steamed cake | 150 | 393.5 |
|  |  | Millet porridge | 200 | 91.0 |
|  | Non-staple food (dishes) | Microwave dishes | 180 | 194.9 |
|  | Non-staple food (dishes) | Pickles / sauce | 15 | 9.0 |
|  | Non-staple food (dishes) | Spicy duck | 80 | 201.8 |
|  | Condiment | Condiment | 10 | / |
|  | Subtotal |  | 635 | 890.2 |
| Refreshments | Ready to eat | Fruit | 100 | 34.2 |
|  |  | Yogurt | 150 | 159.2 |
|  |  | Steamed potatoes | 100 | 109.6 |
|  |  | Chocolate | 14 | 72.7 |
|  | Sum |  | 1944 | 2636 |

**Table S2-14** Recipe No.14: Day47/54

| Meal | Classification | Dishes/item | Weight（g） | Calorie（kcal） |
| --- | --- | --- | --- | --- |
| Breakfast | Staple food | Bread | 175 | 529.1 |
|  | Staple food | Milk | 120 | 107.2 |
|  | Non-staple food (dishes) | Mixing vegetables | 80 | 86.6 |
|  | Non-staple food (dishes) | Sauce | 15 | 9.0 |
|  | Non-staple food (dishes) | Spiced corn and fried eggs | 45 | 69.3 |
|  | Others | Instant spirulina | 10 | 2.8 |
|  | Condiment | Condiment | 10 | 27.0 |
|  | Subtotal |  | 455 | 831 |
| Lunch | Staple food | Noodles | 200 | 470.3 |
|  | Non-staple food (dishes) | Spicy potato strip | 200 | 283.2 |
|  | Non-staple food (dishes) | Pickles / sauce | 15 | 9.0 |
|  | Non-staple food (dishes) | Vegetable soup | 100 | 63.0 |
|  | Non-staple food (dishes) | Roast chicken leg | 80 | 176.5 |
|  | Non-staple food (dishes) | Spicy pork | 40 | 79.1 |
|  | Condiment | Condiment | 10 | / |
|  | Subtotal |  | 645 | 1081.1 |
| Supper | Staple food | Steamed sweet potato | 100 | 108.2 |
|  | Staple food | Dumplings | 200 | 535.2 |
|  | Non-staple food (dishes) | Mixing vegetables | 80 | 86.6 |
|  | Non-staple food (dishes) | Pickles / sauce | 15 | 9.0 |
|  | Non-staple food (included in dumplings) | Spicy pork | 80 | / |
|  | Condiment | Condiment | 10 | / |
|  | Subtotal |  | 485 | 739 |
| Refreshments | Ready to eat | Fruit | 100 | 34.2 |
|  |  | Bean strip | 20 | 53.9 |
|  | Sum |  | 1705 | 2739 |

**Table S2-15** Recipe No.15: Day61/68/75/82/89/96/103/110/117

| Meal | Classification | Dishes/item | Weight（g） | Calorie（kcal） |
| --- | --- | --- | --- | --- |
| Breakfast | Staple food | Bread | 175 | 529.1 |
|  | Staple food | Sweet soy milk | 150.0 | 84.3 |
|  | Non-staple food (dishes) | Mixing vegetables | 80 | 86.6 |
|  | Non-staple food (dishes) | Sauce | 15 | 9.0 |
|  | Non-staple food (dishes) | Spiced corn and fried eggs | 45 | 69.3 |
|  | Others | Instant spirulina | 10 | 2.8 |
|  | Condiment | Condiment | 20 | 54.0 |
|  | Subtotal |  | 495 | 835.1 |
| Lunch | Staple food | Steamed rice | 200 | 235.8 |
|  | Non-staple food (dishes) | Vegetable salad | 180 | 115.3 |
|  | Non-staple food (dishes) | Pickles / sauce | 15 | 9.0 |
|  | Non-staple food (dishes) | Vegetable soup | 100 | 63.0 |
|  | Non-staple food (dishes) | Cabbage stewed rabbit meat | 80 | 134.9 |
|  | Non-staple food (dishes) | Barbecued chicken | 40 | 78.4 |
|  | Condiment | Condiment | 10 | / |
|  | Subtotal |  | 625 | 636.4 |
| Supper | Staple food | Noodles | 200 | 470.3 |
|  | Non-staple food (dishes) | Spicy potato strip | 200 | 283.2 |
|  | Non-staple food (dishes) | Pickles / sauce | 15 | 9.0 |
|  | Non-staple food (dishes) | Vegetable soup | 100 | 63.0 |
|  | Non-staple food (dishes) | Carrot duck | 80 | 113.9 |
|  | Non-staple food (dishes) | Saucebeef | 40 | 62.0 |
|  | Condiment | Condiment | 10 | / |
|  | Subtotal |  | 645 | 1001.4 |
| Refreshments | Ready to eat | Fruit | 100 | 34.2 |
|  |  | Milk | 120.0 | 107.2 |
|  |  | Chocolate | 14 | 72.7 |
|  | Sum |  | 1999 | 2687 |

**Table S2-16** Recipe No.16: Day62/69/76/83/90/97/104/111/118

| Meal | Classification | Dishes/item | Weight（g） | Calorie（kcal） |
| --- | --- | --- | --- | --- |
| Breakfast | Staple food | Flour soup | 200 | 253.1 |
|  | Non-staple food (dishes) | Saltypeanut | 30 | 98.6 |
|  | Non-staple food (dishes) | Mixing vegetables | 80 | 86.6 |
|  | Non-staple food (dishes) | Pickles / sauce | 15 | 9.0 |
|  | Non-staple food (dishes) | Spiced quail eggs | 35 | 96.8 |
|  | Others | Instant spirulina | 10 | 2.8 |
|  | Condiment | Condiment | 10 | 27.0 |
|  | Subtotal |  | 380 | 573.9 |
| Lunch | Staple food | Noodles | 200 | 470.3 |
|  | Non-staple food (dishes) | Vegetable salad | 180 | 115.3 |
|  | Non-staple food (dishes) | Pickles / sauce | 15 | 9.0 |
|  | Non-staple food (dishes) | Vegetable soup | 100 | 63.0 |
|  | Non-staple food (dishes) | Black Pepper Beef | 80 | 134.2 |
|  | Non-staple food (dishes) | Spicy tuna | 40 | 59.2 |
|  | Condiment | Condiment | 10 | / |
|  | Subtotal |  | 625 | 851 |
| Supper | Staple food | Dough piece | 200 | 470.3 |
|  | Non-staple food (dishes) | Microwave dishes | 180 | 194.9 |
|  | Non-staple food (dishes) | Pickles / sauce | 15 | 9.0 |
|  | Non-staple food (dishes) | Vegetable soup | 100 | 63.0 |
|  | Non-staple food (dishes) | Shrimp egg | 80 | 118.2 |
|  | Non-staple food (dishes) | Spicy chicken wings | 40 | 70.4 |
|  | Condiment | Condiment | 10 | / |
|  | Subtotal |  | 625 | 925.8 |
| Refreshments | Ready to eat | Fruit | 100 | 34.2 |
|  |  | Steamed potatoes | 100 | 109.6 |
|  |  | Yogurt | 150 | 159.2 |
|  |  | Chocolate | 14 | 72.7 |
|  | Sum |  | 1994 | 2726 |

**Table S2-17** Recipe No.17: Day63/70/77/84/91/98/105/112/119

| Meal | Classification | Dishes/item | Weight（g） | Calorie（kcal） |
| --- | --- | --- | --- | --- |
| Breakfast | Staple food | Rice porridge | 200 | 91.0 |
|  | Staple food | Steamed sweet potato | 100 | 108.2 |
|  | Non-staple food (dishes) | Mixing vegetables | 80 | 86.6 |
|  | Non-staple food (dishes) | Sauce | 15 | 9.0 |
|  | Non-staple food (dishes) | Spiced corn and fried eggs | 45 | 69.3 |
|  | Others | Instant spirulina | 10 | 2.8 |
|  | Condiment | Condiment | 10 | 27.0 |
|  | Subtotal |  | 460 | 393.9 |
| Lunch | Staple food | Noodles | 200 | 470.3 |
|  | Non-staple food (dishes) | Spicy potato strip | 200 | 283.2 |
|  | Non-staple food (dishes) | Pickles / sauce | 15 | 9.0 |
|  | Non-staple food (dishes) | Vegetable soup | 100 | 63.0 |
|  | Non-staple food (dishes) | Milky chicken corn | 80 | 94.9 |
|  | Non-staple food (dishes) | Pork sausage | 40 | 104.7 |
|  | Condiment | Condiment | 10 | / |
|  | Subtotal |  | 645 | 1025.1 |
| Supper | Staple food | Noodles | 200 | 470.3 |
|  | Non-staple food (dishes) | Microwave dishes | 180 | 194.9 |
|  | Non-staple food (dishes) | Pickles / sauce | 15 | 9.0 |
|  | Non-staple food (dishes) | Vegetable soup | 100 | 63.0 |
|  | Non-staple food (dishes) | Steamed tendon | 80 | 160.7 |
|  | Non-staple food (dishes) | Cardamom | 40 | 83.6 |
|  | Condiment | Condiment | 10 | / |
|  | Subtotal |  | 625 | 981.5 |
| Refreshments | Ready to eat | Fruit | 100 | 34.2 |
|  |  | Yogurt | 150 | 159.2 |
|  |  | soy milk | 150.0 | 41.8 |
|  |  | Chocolate | 14 | 72.7 |
|  | Sum |  | 2130 | 2708 |

**Table S2-18** Recipe No.18: Day64/71/78/85/92/99/106/113/120

| Meal | Classification | Dishes/item | Weight（g） | Calorie（kcal） |
| --- | --- | --- | --- | --- |
| Breakfast | Staple food | Flour soup | 200 | 253.1 |
|  | Non-staple food (dishes) | Saltypeanut | 30 | 98.6 |
|  | Non-staple food (dishes) | Mixing vegetables | 80 | 86.6 |
|  | Non-staple food (dishes) | Pickles / sauce | 15 | 9.0 |
|  | Non-staple food (dishes) | Spiced quail eggs | 35 | 96.8 |
|  | Others | Instant spirulina | 10 | 2.8 |
|  | Condiment | Condiment | 10 | 27.0 |
|  | Subtotal |  | 380 | 573.9 |
| Lunch | Staple food | Noodles | 200 | 470.3 |
|  | Non-staple food (dishes) | Vegetable salad | 180 | 115.3 |
|  | Non-staple food (dishes) | Pickles / sauce | 15 | 9.0 |
|  | Non-staple food (dishes) | Spicy lamb | 80 | 138.7 |
|  | Non-staple food (dishes) | Fragrant chicken | 40 | 60.7 |
|  | Non-staple food (dishes) | Vegetable soup | 100 | 63.0 |
|  | Condiment | Condiment | 10 | / |
|  | Subtotal |  | 625 | 857 |
| Supper | Staple food | Steamed cake | 150 | 393.5 |
|  | Staple food | Millet porridge | 200 | 91.0 |
|  | Non-staple food (dishes) | Microwave dishes | 180 | 194.9 |
|  | Non-staple food (dishes) | Pickles / sauce | 15 | 9.0 |
|  | Non-staple food (dishes) | Edible tree fungusbeef | 80 | 86.6 |
|  | Non-staple food (dishes) | Fragrant pork ear | 40 | 88.4 |
|  | Condiment | Condiment | 10 | / |
|  | Subtotal |  | 675 | 863.4 |
| Refreshments | Ready to eat | Fruit | 100 | 34.2 |
|  |  | Steamed potatoes | 100 | 109.6 |
|  |  | Yogurt | 150 | 159.2 |
|  |  | Chocolate | 14 | 72.7 |
|  | Sum |  | 2044 | 2670 |

**Table S2-19** Recipe No.19: Day65/72/79/86/93/100/107/114

| Meal | Classification | Dishes/item | Weight（g） | Calorie（kcal） |
| --- | --- | --- | --- | --- |
| Breakfast | Staple food | Bread | 175 | 529.1 |
|  | Staple food | Sweet soy milk | 200 | 112.4 |
|  | Non-staple food (dishes) | Mixing vegetables | 80 | 86.6 |
|  | Non-staple food (dishes) | Sauce | 15 | 9.0 |
|  | Non-staple food (dishes) | Spiced corn and fried eggs | 45 | 69.3 |
|  | Others | Instant spirulina | 10 | 2.8 |
|  | Condiment | Condiment | 10 | 27.0 |
|  | Subtotal |  | 535 | 836.2 |
| Lunch | Staple food | Noodles | 200 | 470.3 |
|  | Non-staple food (dishes) | Vegetable salad | 180 | 115.3 |
|  | Non-staple food (dishes) | Pickles / sauce | 15 | 9.0 |
|  | Non-staple food (dishes) | Vegetable soup | 100 | 63.0 |
|  | Non-staple food (dishes) | Bazhen chicken | 80 | 117.7 |
|  | Non-staple food (dishes) | Pingyaobeef | 40 | 61.2 |
|  | Condiment | Condiment | 10 | / |
|  | Subtotal |  | 625 | 836.5 |
| Supper | Staple food | Steamed bread | 150 | 338.9 |
|  | Staple food | Rice porridge | 100 | 46.7 |
|  | Non-staple food (dishes) | Spicy potato strip | 200 | 283.2 |
|  | Non-staple food (dishes) | Pickles / sauce | 15 | 9.0 |
|  | Non-staple food (dishes) | Muxurou | 80 | 122.0 |
|  | Non-staple food (dishes) | Spicy goose | 40 | 67.4 |
|  | Condiment | Condiment | 10 | / |
|  | Subtotal |  | 595 | 867.2 |
| Refreshments | Ready to eat | Fruit | 100 | 34.2 |
|  |  | Chocolate | 14 | 72.7 |
|  | Sum |  | 1869 | 2647 |

**Table S2-20** Recipe No.20: Day66/73/80/87/94/101/108/115

| Meal | Classification | Dishes/item | Weight（g） | Calorie（kcal） |
| --- | --- | --- | --- | --- |
| Breakfast | Staple food | Flour soup | 200 | 253.1 |
|  | Staple food | Steamed sweet potato | 100 | 108.2 |
|  | Non-staple food (dishes) | Saltypeanut | 30 | 98.6 |
|  | Non-staple food (dishes) | Mixing vegetables | 80 | 86.6 |
|  | Non-staple food (dishes) | Pickles / sauce | 15 | 9.0 |
|  | Non-staple food (dishes) | Spiced quail eggs | 35 | 96.8 |
|  | Others | Instant spirulina | 10 | 2.8 |
|  | Condiment | Condiment | 10 | 27.0 |
|  | Subtotal |  | 380 | 682.1 |
| Lunch | Staple food | Noodles | 200 | 470.3 |
|  | Non-staple food (dishes) | Vegetable salad | 180 | 115.3 |
|  | Non-staple food (dishes) | Pickles / sauce | 15 | 9.0 |
|  | Non-staple food (dishes) | Vegetable soup | 100 | 63.0 |
|  | Non-staple food (dishes) | Pork chops | 80 | 177.4 |
|  | Condiment | Condiment | 10 | / |
|  | Subtotal |  | 585 | 835 |
| Supper | Staple food | Steamed cake | 150 | 393.5 |
|  |  | Millet porridge | 200 | 91.0 |
|  | Non-staple food (dishes) | Microwave dishes | 180 | 194.9 |
|  | Non-staple food (dishes) | Pickles / sauce | 15 | 9.0 |
|  | Non-staple food (dishes) | Spicy duck | 80 | 201.8 |
|  | Condiment | Condiment | 10 | / |
|  | Subtotal |  | 635 | 890.2 |
| Refreshments | Ready to eat | Fruit | 100 | 34.2 |
|  |  | Yogurt | 150 | 159.2 |
|  |  | Steamed potatoes | 100 | 109.6 |
|  |  | Chocolate | 14 | 72.7 |
|  | Sum |  | 2064 | 2783 |

**Table S2-21** Recipe No.21: Day67/74/81/88/95/102/109/116

| Meal | Classification | Dishes/item | Weight（g） | Calorie（kcal） |
| --- | --- | --- | --- | --- |
| Breakfast | Staple food | Bread | 175 | 529.1 |
|  | Staple food | Sweet soy milk | 200 | 112.4 |
|  | Non-staple food (dishes) | Mixing vegetables | 80 | 86.6 |
|  | Non-staple food (dishes) | Sauce | 15 | 9.0 |
|  | Non-staple food (dishes) | Spiced corn and fried eggs | 45 | 69.3 |
|  | Others | Instant spirulina | 10 | 2.8 |
|  | Condiment | Condiment | 10 | 27.0 |
|  | Subtotal |  | 535 | 836.2 |
| Lunch | Staple food | Noodles | 200 | 470.3 |
|  | Non-staple food (dishes) | Spicy potato strip | 200 | 283.2 |
|  | Non-staple food (dishes) | Pickles / sauce | 15 | 9.0 |
|  | Non-staple food (dishes) | Vegetable soup | 100 | 63.0 |
|  | Non-staple food (dishes) | Roast chicken leg | 80 | 176.5 |
|  | Non-staple food (dishes) | Spicy pork | 40 | 79.1 |
|  | Condiment | Condiment | 10 | / |
|  | Subtotal |  | 645 | 1081.1 |
| Supper | Staple food | Steamed sweet potato | 100 | 108.2 |
|  | Staple food | Dumplings | 200 | 535.2 |
|  | Non-staple food (dishes) | Mixing vegetables | 80 | 86.6 |
|  | Non-staple food (dishes) | Pickles / sauce | 15 | 9.0 |
|  | Non-staple food (included in dumplings) | Spicy pork | 80 | / |
|  | Condiment | Condiment | 10 | / |
|  | Subtotal |  | 485 | 739 |
| Refreshments | Ready to eat | Fruit | 100 | 34.2 |
|  | Sum |  | 1765 | 2691 |

**Table S2-22 Recipe No.22: Day121/128/135/142/149/156/163/170/177**

| Meal | Classification | Dishes/item | Weight（g） | Calorie（kcal） |
| --- | --- | --- | --- | --- |
| Breakfast | Staple food | Bread | 175 | 529.1 |
|  | Staple food | Sweet soy milk | 150 | 84.3 |
|  | Non-staple food (dishes) | Mixing vegetables | 80 | 86.6 |
|  | Non-staple food (dishes) | Sauce | 15 | 9.0 |
|  | Non-staple food (dishes) | Spiced corn and fried eggs | 45 | 69.3 |
|  | Others | Instant spirulina | 10 | 2.8 |
|  | Condiment | Condiment | 20 | 54.0 |
|  | Subtotal |  | 495 | 835.1 |
| Lunch | Staple food | Steamed rice | 200 | 235.8 |
|  | Non-staple food (dishes) | Vegetable salad | 180 | 115.3 |
|  | Non-staple food (dishes) | Pickles / sauce | 15 | 9.0 |
|  | Non-staple food (dishes) | Vegetable soup | 100 | 63.0 |
|  | Non-staple food (dishes) | Cabbage stewed rabbit meat | 80 | 134.9 |
|  | Non-staple food (dishes) | Barbecued chicken | 40 | 78.4 |
|  | Condiment | Condiment | 10 | / |
|  | Subtotal |  | 625 | 636.4 |
| Supper | Staple food | Noodles | 200 | 470.3 |
|  | Non-staple food (dishes) | Spicy potato strip | 200 | 283.2 |
|  | Non-staple food (dishes) | Pickles / sauce | 15 | 9.0 |
|  | Non-staple food (dishes) | Vegetable soup | 100 | 63.0 |
|  | Non-staple food (dishes) | Carrot duck | 80 | 113.9 |
|  | Non-staple food (dishes) | Saucebeef | 40 | 62.0 |
|  | Condiment | Condiment | 10 | / |
|  | Subtotal |  | 645 | 1001.4 |
| Refreshments | Ready to eat | Fruit | 100 | 34.2 |
|  |  | Milk | 120 | 107.2 |
|  |  | Chocolate | 14 | 72.7 |
|  | Sum |  | 2000 | 2687 |

**Table S2-23** Recipe No.23: Day122/129/136/143/150/157/164/171/178

| Meal | Classification | Dishes/item | Weight（g） | Calorie（kcal） |
| --- | --- | --- | --- | --- |
| Breakfast | Staple food | Flour soup | 200 | 253.1 |
|  | Non-staple food (dishes) | Saltypeanut | 30 | 98.6 |
|  | Non-staple food (dishes) | Mixing vegetables | 80 | 86.6 |
|  | Non-staple food (dishes) | Pickles / sauce | 15 | 9.0 |
|  | Non-staple food (dishes) | Spiced quail eggs | 35 | 96.8 |
|  | Others | Instant spirulina | 10 | 2.8 |
|  | Condiment | Condiment | 10 | 27.0 |
|  | Subtotal |  | 380 | 573.9 |
| Lunch | Staple food | Noodles | 200 | 470.3 |
|  | Non-staple food (dishes) | Vegetable salad | 180 | 115.3 |
|  | Non-staple food (dishes) | Pickles / sauce | 15 | 9.0 |
|  | Non-staple food (dishes) | Vegetable soup | 100 | 63.0 |
|  | Non-staple food (dishes) | Potato beef | 80 | 120 |
|  | Non-staple food (dishes) | Spiced squid | 40 | 54.7 |
|  | Condiment | Condiment | 10 | / |
|  | Subtotal |  | 625 | 832.3 |
| Supper | Staple food | Dough piece | 200 | 470.3 |
|  | Non-staple food (dishes) | Microwave dishes | 180 | 194.9 |
|  | Non-staple food (dishes) | Pickles / sauce | 15 | 9.0 |
|  | Non-staple food (dishes) | Vegetable soup | 100 | 63.0 |
|  | Non-staple food (dishes) | Shrimp egg | 80 | 118.2 |
|  | Non-staple food (dishes) | Spicy chicken wings | 40 | 70.4 |
|  | Condiment | Condiment | 10 | / |
|  | Subtotal |  | 625 | 925.8 |
| Refreshments | Ready to eat | Fruit | 100 | 34.2 |
|  |  | Steamed potatoes | 100 | 109.6 |
|  |  | Yogurt | 150 | 159.2 |
|  |  | Chocolate | 14 | 72.7 |
|  | Sum |  | 1994 | 2708 |

**Table S2-24** Recipe No.24: Day123/130/137/144/151/158/165/172/179

| Meal | Classification | Dishes/item | Weight（g） | Calorie（kcal） |
| --- | --- | --- | --- | --- |
| Breakfast | Staple food | Rice porridge | 200 | 91.0 |
|  | Staple food | Steamed sweet potato | 100 | 108.2 |
|  | Non-staple food (dishes) | Mixing vegetables | 80 | 86.6 |
|  | Non-staple food (dishes) | Sauce | 15 | 9.0 |
|  | Non-staple food (dishes) | Spiced corn and fried eggs | 45 | 69.3 |
|  | Others | Instant spirulina | 10 | 2.8 |
|  | Condiment | Condiment | 10 | 27.0 |
|  | Subtotal |  | 460 | 393.9 |
| Lunch | Staple food | Noodles | 200 | 470.3 |
|  | Non-staple food (dishes) | Spicy potato strip | 200 | 283.2 |
|  | Non-staple food (dishes) | Pickles / sauce | 15 | 9.0 |
|  | Non-staple food (dishes) | Vegetable soup | 100 | 63.0 |
|  | Non-staple food (dishes) | Carrot duck | 80 | 113.9 |
|  | Non-staple food (dishes) | Pork sausage | 40 | 104.7 |
|  | Condiment | Condiment | 10 | / |
|  | Subtotal |  | 645 | 1034.1 |
| Supper | Staple food | Noodles | 200 | 470.3 |
|  | Non-staple food (dishes) | Microwave dishes | 180 | 194.9 |
|  | Non-staple food (dishes) | Pickles / sauce | 15 | 9.0 |
|  | Non-staple food (dishes) | Vegetable soup | 100 | 63.0 |
|  | Non-staple food (dishes) | Steamed tendon | 80 | 160.7 |
|  | Non-staple food (dishes) | Cardamom | 40 | 83.6 |
|  | Condiment | Condiment | 10 | / |
|  | Subtotal |  | 625 | 981.5 |
| Refreshments | Ready to eat | Fruit | 100 | 34.2 |
|  |  | Yogurt | 150 | 159.2 |
|  |  | Saltygreen soybean / soy milk | 150 | 41.8 |
|  |  | Chocolate | 14 | 72.7 |
|  | Sum |  | 2144 | 2727 |

**Table S2-25** Recipe No.25: Day124/131/138/145/152/159/166/173/180

| Meal | Classification | Dishes/item | Weight（g） | Calorie（kcal） |
| --- | --- | --- | --- | --- |
| Breakfast | Staple food | Flour soup | 200 | 253.1 |
|  | Non-staple food (dishes) | Saltypeanut | 30 | 98.6 |
|  | Non-staple food (dishes) | Mixing vegetables | 80 | 86.6 |
|  | Non-staple food (dishes) | Pickles / sauce | 15 | 9.0 |
|  | Non-staple food (dishes) | Spiced quail eggs | 35 | 96.8 |
|  | Others | Instant spirulina | 10 | 2.8 |
|  | Condiment | Condiment | 10 | 27.0 |
|  | Subtotal |  | 380 | 573.9 |
| Lunch | Staple food | Noodles | 200 | 470.3 |
|  | Non-staple food (dishes) | Vegetable salad | 180 | 115.3 |
|  | Non-staple food (dishes) | Pickles / sauce | 15 | 9.0 |
|  | Non-staple food (dishes) | Vegetable soup | 100 | 63.0 |
|  | Non-staple food (dishes) | Cabbage stewed rabbit meat | 80 | 134.9 |
|  | Non-staple food (dishes) | Fragrant chicken | 40 | 60.7 |
|  | Condiment | Condiment | 10 | / |
|  | Subtotal |  | 625 | 853.2 |
| Supper | Staple food | Steamed cake | 150 | 393.5 |
|  | Staple food | Millet porridge | 200 | 91.0 |
|  | Non-staple food (dishes) | Microwave dishes | 180 | 194.9 |
|  | Non-staple food (dishes) | Pickles / sauce | 15 | 9.0 |
|  | Non-staple food (dishes) | Shrimp egg | 80 | 118.2 |
|  | Non-staple food (dishes) | Fragrant pork ear | 40 | 88.4 |
|  | Condiment | Condiment | 10 | / |
|  | Subtotal |  | 675 | 895 |
| Refreshments | Ready to eat | Fruit | 100 | 34.2 |
|  |  | Steamed potatoes | 100 | 109.6 |
|  |  | Yogurt | 150 | 159.2 |
|  |  | Chocolate | 14 | 72.7 |
|  | Sum |  | 2044 | 2698 |

**Table S2-26** Recipe No.26: Day125/132/139/146/153/160/167/174

| Meal | Classification | Dishes/item | Weight（g） | Calorie（kcal） |
| --- | --- | --- | --- | --- |
| Breakfast | Staple food | Bread | 175 | 529.1 |
|  | Staple food | Sweet soy milk | 200 | 112.4 |
|  | Non-staple food (dishes) | Mixing vegetables | 80 | 86.6 |
|  | Non-staple food (dishes) | Sauce | 15 | 9.0 |
|  | Non-staple food (dishes) | Spiced corn and fried eggs | 45 | 69.3 |
|  | Others | Instant spirulina | 10 | 2.8 |
|  | Condiment | Condiment | 10 | 27.0 |
|  | Subtotal |  | 535 | 836.2 |
| Lunch | Staple food | Noodles | 200 | 470.3 |
|  | Non-staple food (dishes) | Vegetable salad | 180 | 115.3 |
|  | Non-staple food (dishes) | Pickles / sauce | 15 | 9.0 |
|  | Non-staple food (dishes) | Vegetable soup | 100 | 63.0 |
|  | Non-staple food (dishes) | Spicy pork | 80 | 121.2 |
|  | Non-staple food (dishes) | Pingyaobeef | 40 | 61.2 |
|  | Condiment | Condiment | 10 | / |
|  | Subtotal |  | 625 | 840.0 |
| Supper | Staple food | Steamed bread | 150 | 338.9 |
|  | Staple food | Rice porridge | 100 | 46.7 |
|  | Non-staple food (dishes) | Spicy potato strip | 200 | 283.2 |
|  | Non-staple food (dishes) | Pickles / sauce | 15 | 9.0 |
|  | Non-staple food (dishes) | Potato beef | 80 | 120.0 |
|  | Non-staple food (dishes) | Spicy goose | 40 | 67.4 |
|  | Condiment | Condiment | 10 | / |
|  | Subtotal |  | 595 | 865.2 |
| Refreshments | Ready to eat | Fruit | 100 | 34.2 |
|  |  | Chocolate | 14 | 72.7 |
|  | Sum |  | 1879 | 2648 |

**Table S2-27** Recipe No.27: Day126/133/140/147/154/161/168/175

| Meal | Classification | Dishes/item | Weight（g） | Calorie（kcal） |
| --- | --- | --- | --- | --- |
| Breakfast | Staple food | Flour soup | 200 | 253.1 |
|  | Staple food | Steamed sweet potato | 100 | 108.2 |
|  | Non-staple food (dishes) | Salty peanut | 30 | 98.6 |
|  | Non-staple food (dishes) | Mixing vegetables | 80 | 86.6 |
|  | Non-staple food (dishes) | Pickles / sauce | 15 | 9.0 |
|  | Non-staple food (dishes) | Spiced quail eggs | 35 | 96.8 |
|  | Others | Instant spirulina | 10 | 2.8 |
|  | Condiment | Condiment | 10 | 27.0 |
|  | Subtotal |  | 380 | 682.1 |
| Lunch | Staple food | Noodles | 200 | 470.3 |
|  | Non-staple food (dishes) | Vegetable salad | 180 | 115.3 |
|  | Non-staple food (dishes) | Pickles / sauce | 15 | 9.0 |
|  | Non-staple food (dishes) | Vegetable soup | 100 | 63.0 |
|  | Non-staple food (dishes) | Pork chops | 80 | 177.4 |
|  | Condiment | Condiment | 10 | / |
|  | Subtotal |  | 585 | 835 |
| Supper | Staple food | Steamed cake | 150 | 393.5 |
|  |  | Millet porridge | 200 | 91.0 |
|  | Non-staple food (dishes) | Microwave dishes | 180 | 194.9 |
|  | Non-staple food (dishes) | Pickles / sauce | 15 | 9.0 |
|  | Non-staple food (dishes) | Spicy duck | 80 | 201.8 |
|  | Condiment | Condiment | 10 | / |
|  | Subtotal |  | 635 | 890.2 |
| Refreshments | Ready to eat | Fruit | 100 | 34.2 |
|  |  | Yogurt | 150 | 159.2 |
|  |  | Steamed potatoes | 100 | 109.6 |
|  |  | Chocolate | 14 | 72.7 |
|  | Sum |  | 2064 | 2710 |

**Table S2-28** Recipe No.28: Day127/134/141/148/155/162/169/176

| Meal | Classification | Dishes/item | Weight（g） | Calorie（kcal） |
| --- | --- | --- | --- | --- |
| Breakfast | Staple food | Bread | 175 | 529.1 |
|  | Staple food | Sweet soy milk | 200 | 112.4 |
|  | Non-staple food (dishes) | Mixing vegetables | 80 | 86.6 |
|  | Non-staple food (dishes) | Sauce | 15 | 9.0 |
|  | Non-staple food (dishes) | Spiced corn and fried eggs | 45 | 69.3 |
|  | Others | Instant spirulina | 10 | 2.8 |
|  | Condiment | Condiment | 10 | 27.0 |
|  | Subtotal |  | 535 | 836.2 |
| Lunch | Staple food | Noodles | 200 | 470.3 |
|  | Non-staple food (dishes) | Spicy potato strip | 200 | 283.2 |
|  | Non-staple food (dishes) | Pickles / sauce | 15 | 9.0 |
|  | Non-staple food (dishes) | Vegetable soup | 100 | 63.0 |
|  | Non-staple food (dishes) | Steamed tendon | 80 | 330.4 |
|  | Non-staple food (dishes) | Spicy pork | 40 | 79.1 |
|  | Condiment | Condiment | 10 | 0.0 |
|  | Subtotal |  | 645 | 1081.1 |
| Supper | Staple food | Steamed sweet potato | 100 | 108.2 |
|  | Staple food | Dumplings | 200 | 535.2 |
|  | Non-staple food (dishes) | Mixing vegetables | 80 | 86.6 |
|  | Non-staple food (dishes) | Pickles / sauce | 15 | 9.0 |
|  | Non-staple food (included in dumplings) | Spicy pork | 80 | / |
|  | Condiment | Condiment | 10 | / |
|  | Subtotal |  | 485 | 739 |
| Refreshments | Ready to eat | Fruit | 100 | 34.2 |
|  | Sum |  | 1765 | 2845 |
